# Socioecological correlates of parental lifestyle patterns during the antenatal period

**DOI:** 10.1186/s12966-024-01697-1

**Published:** 2025-02-13

**Authors:** M. Lecorguillé, M. C. Schipper, A. M. Aubert, A. Douglass, M. Tafflet, M. Vrijheid, C. Kelleher, C. M. Phillips, R. Gaillard, Barbara Heude, Sandrine Lioret

**Affiliations:** 1 Center for Research in Epidemiology and StatisticS (CRESS), Université Paris Cité and Université Sorbonne Paris Nord, Inserm, INRAE, Paris, F-75004 France; 2 The Generation R Study Group (Na 29-15), Erasmus University Medical Center, PO Box 2040, Rotterdam, CA 3000 The Netherlands; 3 Department of Paediatrics, Erasmus MC-University Medical Centre Rotterdam, Rotterdam, Netherlands; 4 School of Public Health, Physiotherapy and Sports Science, University College Dublin, Dublin, Republic of Ireland; 5 ISGlobal, Barcelona, Spain; 6 Universitat Pompeu Fabra (UPF), Barcelona, Spain; 7 Spanish Consortium for Research on Epidemiology and Public Health (CIBERESP), Madrid, Spain

**Keywords:** 1000 days, Parental lifestyle patterns, Pregnancy, Childhood obesity, Social determinants, Socioecological model, Urban environment

## Abstract

**Background:**

This study aimed to explore socioecological correlates of parental lifestyle patterns during pregnancy, an overlooked topic except for individual socioeconomic factors.

**Methods:**

We used data from three European mother-offspring cohorts participating in the EndObesity Consortium [EDEN, France, n = 1,962; Generation R, the Netherlands, n = 8,765; and Lifeways, Ireland, n = 932]. In previous principal component analysis, we identified two separate parental lifestyle patterns in pregnancy, characterised by: 1) “high parental smoking, poor-quality maternal diet, and low physical activity”; and 2) “low parental body mass index (BMI) and high gestational weight gain (GWG)”. Applying the socioecological model, we conducted multivariable linear regression analyses on lifestyle pattern scores (outcomes), first including parental socioeconomic and sociodemographic characteristics (block 1), then the urban environment (block 2), and finally psychosocial factors and health-care access (block 3).

**Results:**

Older parents, those born abroad, or with high SEP had lower scores for the first lifestyle pattern. Conversely, multiparous mothers, those with suboptimal health insurance coverage, or who did not attend parenting preparation sessions followed that pattern more closely. Multiparous mothers, parents with a low SEP, or living in highly deprived areas had lower scores on the second pattern, contrary to those exposed to high population density or living in a neighbourhood with high facility richness.

**Conclusions:**

Higher SEP, a foreign birthplace, wealthier neighbourhoods, and attendance at antenatal parenting preparation sessions were associated with healthier parental lifestyles during pregnancy. These potential facilitators should be considered for inclusion in tailored family-based health promotion interventions during the perinatal period.

**Supplementary Information:**

The online version contains supplementary material available at 10.1186/s12966-024-01697-1.

## Background

Childhood overweight or obesity (OW/OB) has reached alarming rates worldwide, affecting about 40 million children younger than 5 years [1]. The strong inverse socioeconomic gradient of childhood overweight observed from early life [2, 3] makes this a major public health and social justice issue. Children with OW/OB are at risk of both maintaining their excess weight into adulthood and developing non-communicable diseases [4].

The high prevalence of OW/OB observed in young children highlights the involvement of early exposures or stressors [5–8]. We previously showed that high body mass index (BMI), smoking, low-quality diet, low physical activity (PA) levels, and regular sedentary behaviours in mothers and fathers during pregnancy, when combined into lifestyle patterns, were associated with a high risk of obesity in children aged 5–12 years [9].

Parental lifestyle behaviours, because they are potentially modifiable, represent important targets for family-based, multi-behavioural child obesity prevention strategies in early life.

The socioecological model proposed by Bronfenbrenner supports the idea that individual characteristics cannot be effectively explained without consideration of the contexts, or ecological niches, in which a person is embedded [10]. Health determinants include each person’s individual characteristics and behaviours, which in turn are influenced by broader physical, social, and economic factors [11]. A low socioeconomic position (SEP, often defined based on education, income, occupation, or some combination of them) is known to be associated with some antenatal risk factors, including but not limited to high maternal prepregnancy BMI, heavy tobacco consumption, and poorer diet quality [12]. The environmental influence on parental lifestyle includes SEP and other social determinants. Targeting groups at higher risk of suboptimal behaviours requires disentangling the social determinants associated with these lifestyle patterns. This approach has proved useful over the past decade for developing public health programmes focused less on individuals and more on the structural determinants shaping their behaviour [13].

Our aim was therefore to investigate the socioecological correlates of parental lifestyle patterns in pregnancy, including socioeconomic and sociodemographic factors, the role of the urban environment, and the influence of psychosocial factors and health-care access. Accordingly, we performed cohort-specific analyses in three European cohorts by harmonising the data and analytic approach.

## Methods

This study is reported according to the Strengthening the Reporting of Observational Studies in Epidemiology (STROBE) guideline (S1 STROBE guideline checklist).

### Study population

**Fig. 1:**
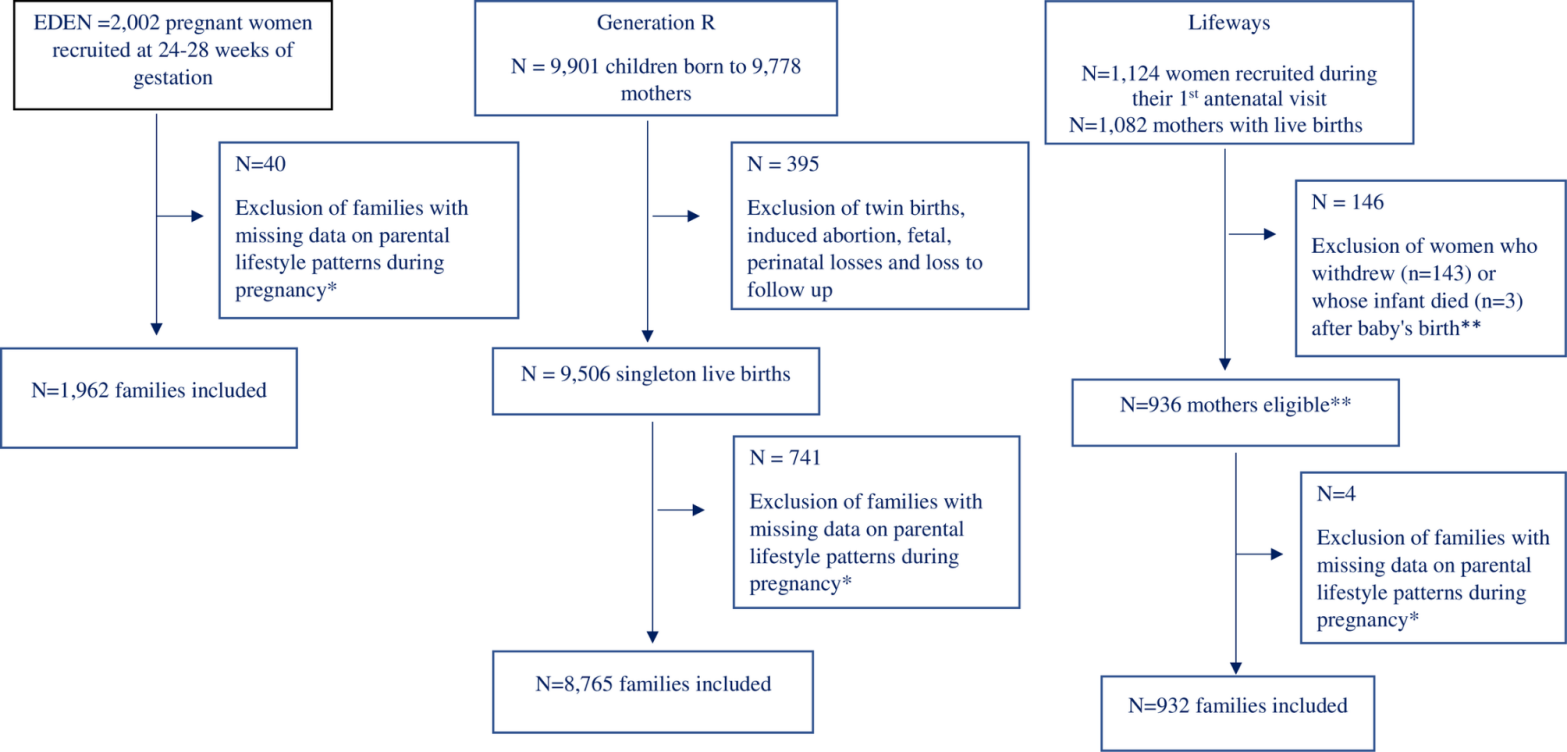
Flow-chart of the selection among populations for imputation and analyses. *Parental lifestyle patterns were imputed as described in the manuscript. **And retaining randomly one out of each pair of twins (*n* = 10)

This project involves three birth cohort studies in three European countries participating in the EndObesity consortium [14] and the EU Child cohort network [15]. It includes the EDEN study on the antenatal and early postnatal determinants of child health and development (recruitment: ≈2,000 pregnant women from Jan 27, 2003, to March 6, 2006) in France; the Generation R Study (recruitment: ≈9,800 pregnant women with delivery expected between April 1, 2002, and Jan 31, 2006) in the Netherlands; and the Lifeways Cross-Generation Cohort Study (recruitment: ≈1,100 pregnant women from Oct 2, 2001, to April 4, 2003) in Ireland (Supplementary Table 1). Parents with multiple pregnancies were excluded in Generation R because of its higher percentage of missing data, and we randomly excluded one twin from each twin pair in Lifeways. Figure 1 presents the flowchart of the final selected populations. The study design for each cohort has been described in previous publications [16–19].

### Ethics committee approval

All participating cohorts obtained the relevant institutional ethics approvals as well as written consents from all families, and research to date has been conducted according to the Declaration of Helsinki guidelines (Supplementary Table 1).

### Parental lifestyle patterns

In a previous work conducted as part of EndObesity [9], we used principal component analysis (PCA) to derive various parental lifestyle patterns during pregnancy [20]. We included in the PCA antenatal lifestyle factors shown to be associated with childhood overweight in the literature, i.e., maternal prepregnancy BMI, paternal BMI at inclusion, parental smoking during pregnancy (none, < 10 cig/day, ≥ 10 cig/day), parental diet quality, maternal GWG, and both parental PA and sedentary behaviours when available. Data were collected using self-administered health and lifestyle questionnaires completed at inclusion or at birth, face-to-face interviews, and information extracted from medical records. Self-reported pre-pregnancy weight and height were used to calculate BMI, serving as a marker of an obesogenic lifestyle due to its association with other health behaviours. Gestational weight gain was calculated as the measured weight at the end of pregnancy (third trimester in Generation R) minus the weight at conception, as reported by mothers. Diet quality was assessed with the dietary approach to stop hypertension (DASH) score [21], and dietary inflammatory potential with the energy-adjusted dietary inflammatory index (E-DII) [22, 23]. Physical activity levels and sedentary behaviours included leisure, sport or occupational activities; however, the questionnaires varied across cohorts, as described in our previous publication [9]Among the various lifestyle patterns identified, we retained those that were both consistent across cohorts and related to the risk of OW/OB in children aged 5–12 years. The first pattern was characterised by “high parental smoking, poor-quality maternal diet, and low maternal leisure PA” in EDEN; “high parental smoking and poor-quality maternal diet” in Generation R; and “high parental smoking, inflammatory diet, low maternal DASH, and rather low paternal PA” in Lifeways. The second pattern was defined by weight status and reported in EDEN and Generation R as “low parental BMI and high GWG” [9]. A physiological inverse relationship between pre-pregnancy BMI and GWG has been well- documented in the literature [24]. Consistent with the 2009 Institute of Medicine guidelines [25], women with a lower BMI at conception generally tend to have higher GWG compared to those with overweight or obesity. The “Low parental BMI, high GWG” pattern, primarily driven by a healthy BMI status, is therefore considered optimal. Reciprocally, a lower score in this pattern reflects a higher parental BMI and lower GWG, which is considered suboptimal.

We also replicated the PCA analyses including maternal factors only, which produced coherent lifestyle patterns: “low smoking and high-quality diet and leisure PA” in EDEN, “high BMI, smoking, and poor-quality diet” in Generation R, and “smoking and poor-quality diet” in Lifeways as first patterns. The second patterns were labelled “low BMI and high GWG” in EDEN, “low BMI, high GWG, and smoking” in Generation R, and “low BMI and high PA” in Lifeways [9]. Lifeways did not collect information on GWG. Scores were calculated for each mother-father pair and for each mother: the higher the score, the more closely they or she fit the pattern.

### Socioecological correlates

**Fig. 2:**
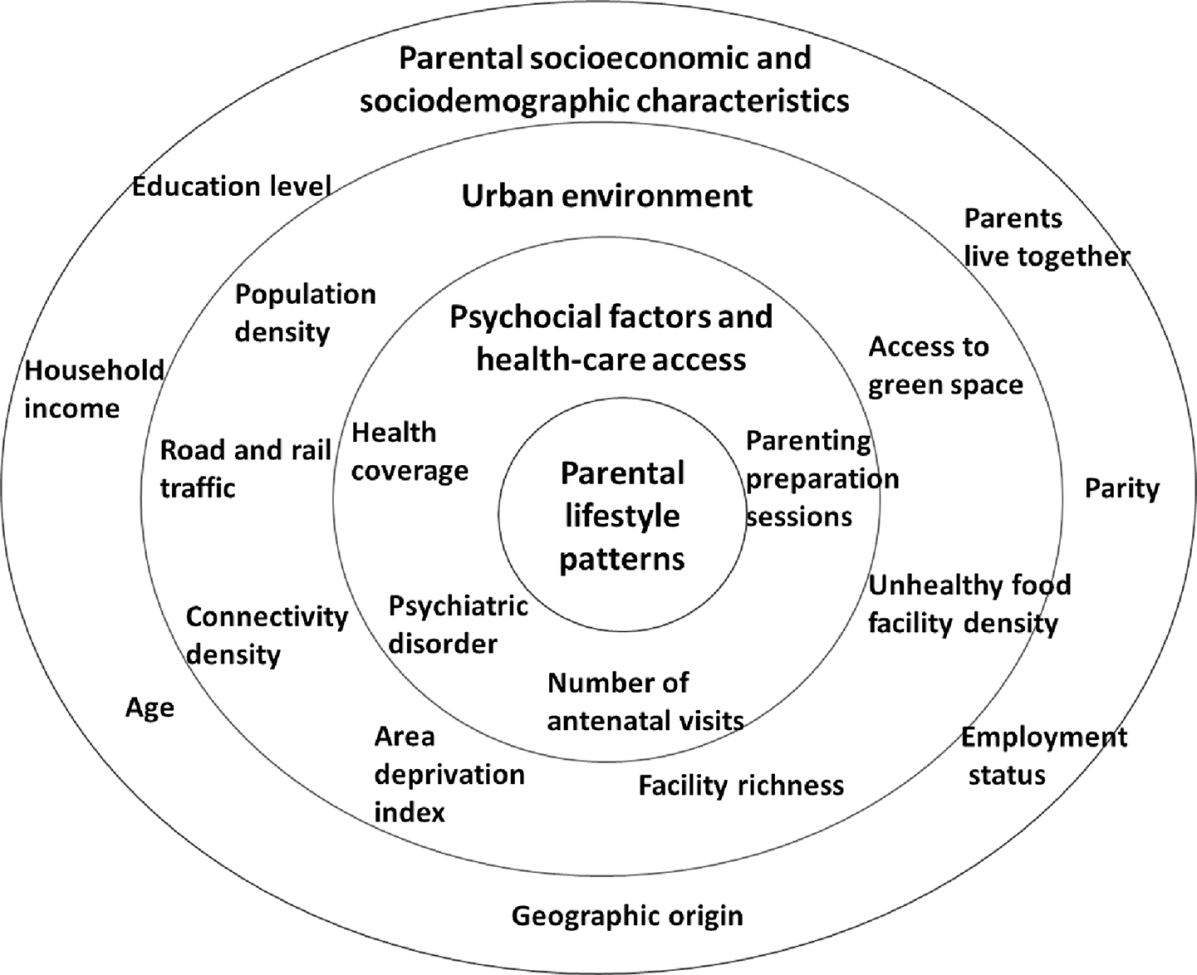
Conceptual framework of contextual factors potentially associated with parental lifestyle patterns during pregnancy

The identification of potential socioecological correlates was based on the factors shown to be associated in the literature with parental individual health factors and their availability in the cohorts at baseline [12, 26–28]. These candidate factors were structured from the most distal to the most proximal to parental lifestyle patterns within a three-nested block framework (Fig. 2), derived from both socioecological [10, 13] and hierarchical [29] approaches. They were organised as follows: parental socioeconomic and sociodemographic characteristics (block 1), urban environment (block 2, not available in Lifeways), and psychosocial factors and health care access (block 3). These three levels of factors were categorised under the assumption that SEP influences the physical environment where families live, which, in turn, influences access to health care and individual factors. We conducted a data inventory and wherever possible we used harmonised variables created within the LifeCycle project [30], a Horizon 2020-funded international project. Protocols for LifeCycle harmonisation are available online at no cost [15]. Supplementary Table 2 summarises the definitions, collection, coding, and harmonisation of these variables of interest.

Briefly, block 1 includes the following variables: parents’ ages, education levels, employment status, birthplace, maternal parity, cohabitation status, and household income. These data were collected by questionnaires (interviewer- or self-administered). In EDEN, we also considered the centre variable (Nancy/Poitiers), a factor that can be associated with family physical environment and health behaviours.

Factors included in block 2 were the urban environment, comprising green space, traffic, and the built environment, which in turn includes markers (all measured within a 300-m buffer) such as street connectivity density, facility richness (defined as all points of interest for pedestrians as part of their daily life activities, e.g., restaurants, shops, medical centres, community services, schools, financial institutions, entertainment, schools, libraries, etc.), the facility richness index (number of different facility types present divided by the maximum potential number of types specified), and density of unhealthy food facilities. Further variables considered in block 2 were population density and the social deprivation index. These last variables were generated for the residential address during pregnancy by using standardised geographic information system protocols developed in the LifeCycle project. Harmonised information on the urban environment was not available for Lifeways. Continuous variables were standardised to interpret the strength of their association with the same unit.

Finally, for psychosocial factors and access to healthcare (block 3), we used information related to maternal mental health during pregnancy; number of antenatal visits; attendance at specific antenatal parenting preparation sessions; and maternal health insurance coverage. Because information was not collected identically in each cohort, it was not possible to harmonise all variables. In the Netherlands, which has a system of mandatory health insurance, all residents generally have access to health care, and this variable was not relevant there.

### Statistical analyses

**Fig. 3:**
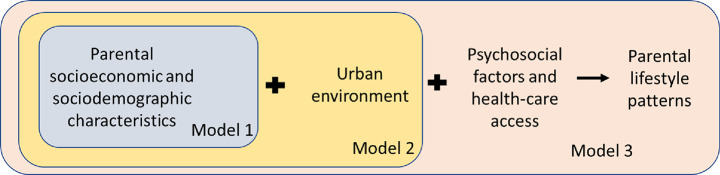
Hierarchical linear regression models

Characteristics of the population with available information on parental lifestyle patterns in pregnancy are described with their means ± SD and percentages (N). Hierarchical linear regression analyses examined the associations between socioecological factors (independent variables) and parental lifestyle patterns (dependent variables), within the three-block socioecological model described above [29] (Fig. 3). Specific estimates were obtained for each cohort. Variables were added per block, from the most distal to the most proximal, and the association coefficients interpreted within the first model in which it was included. This method ensures that intermediate variables (potential mediators) do not affect the associations between distal factors and the dependent variables allowing us to properly interpret total effects. Multicollinearity in the final multivariable model was assessed by inspecting the variance inflation factor (with the threshold of collinearity > 3). Missing data for the socioecological correlates were imputed with the “MICE” R package, which imputes incomplete multivariate data by chained equations. We generated 20 imputed datasets, using logistic and multinomial logit regression models, and predictive mean matching for categorical and quantitative variables [31] (see Supplementary Tables 3–5). Additionally, when evaluating the association with maternal lifestyle patterns during pregnancy, we considered only maternal socioeconomic and sociodemographic characteristics, but we added a minimum adjustment for paternal education level. Lastly, as a sensitivity analysis, we replicated analyses of the third model excluding premature births, on the hypothesis that they may influence follow-up indicators during pregnancy (the number of antenatal visits, for example), and performed analyses on complete cases.

## Results

Table 1 summarises the characteristics and numbers of participants included in this analysis. Across cohorts, substantial percentages of mothers and fathers had attained a high education level (43–54% and 41–50% respectively). Mothers had a mean BMI between 23 and 24 kg/m^2^, and fathers between 25 and 26 kg/m^2^. The prevalence of smoking during pregnancy ranged from 14 to 22% for mothers, and 32–40% for fathers. The percentage of families living in a highly deprived area was 19% in EDEN and 9% in Generation R. Overall, 74–88% of families adhered to the recommended number of antenatal visits, and 36% of the women in EDEN attended all recommended parenting preparation sessions.

**Table 1:** Characteristics of the cohort populations*

	EDEN* (*N* = 1,962)	Gen R (*N* = 8,765)	Lifeways (*N* = 932)
	Population % (*n*) or mean +/- SD	Population % (*n*) or mean +/- SD	Population % (*n*) or mean +/- SD
**Maternal characteristics**			
Maternal age (years)	29.5 ± 4.9	30.3 ± 5.3	29.6 ± 5.9
Born abroad	4.1 (79)	34.3 (2897)	*NA*
Employed/self-employed	76.3 (1461)	72.9 (4772)	67.2 (620)
Maternal education			
Low	7.5 (144)	11.0 (901)	18.8 (171)
Medium	39 (745)	46.0 (3753)	31.3 (285)
High	53.5 (1021)	43.0 (3508)	49.9 (454)
Household income			
1^st^ quartile (lowest)	16.7 (318)	20.1 (1306) ^a^	62.9 (531) ^d^
2^nd^ quartile	29.7 (564)	25.0 (1621) ^b^	37.1 (313) ^e^
3^rd^ quartile	26.3 (500)	54.9 (3563) ^c^	*NA*
4^th^ quartile (highest)	27.2 (517)	*NA*	*NA*
Primiparous	44.6 (848)	56.03 (4810)	45.9 (421)
Maternal pre-pregnancy BMI (kg/m²)	23.28 ± 4.6	23.70 ± 4.39	23.7 ± 4.1
Parents live together	94.1 (1806)	85.80 (7029)	73.7 (686)
Maternal smoking during pregnancy	14 (228)	17.96 (1290)	22.4 (199)
**Paternal characteristics**			
Paternal age (years)	32.0 ± 5.9	33.4 ± 6.0	32.1 ± 6.2
Born abroad	7.3 (138)	35.6 (2785)	*NA*
Employed/self-employed	91.1 (1709)	91.3 (4356)	99.1 (751)
Paternal education			
Low	10 (191)	8.2 (423)	31.8 (261)
Medium	46.3 (885)	41.2 (2115)	26.9 (221)
High	43.7 (835)	50.6 (2602)	41.3 (339)
Paternal prepregnancy BMI (kg/m²)	25 ± 3.6	25.3 ± 3.5	26.6 ± 4.1
Paternal smoking during pregnancy	40.2 (697)	44.8 (3282)	32.3 (87)
**Environmental Variables**			
Population density	2709.8 ± 2231.3	3867.9 ± 638.4	*NA*
Access to green space	136.02 ± 142.1	201.8 ± 160.8	*NA*
Road and rail traffic	9.7 ± 4.6	19.4 ± 5.8	*NA*
Street connectivity density	106.3 ± 65.3	227.6 ± 86.5	*NA*
Food facility density			*NA*
Slightly unhealthy food environment (score 0)	68.3 (1297)	21.4 (1640)	*NA*
Highly unhealthy food environment (score 1)	31.7 (601)	78.6 (6034)	*NA*
Facility richness		0.13 ± 0.1 ^f^	*NA*
0	34 (646)	*NA*	*NA*
0-0.05	34 (646)	*NA*	*NA*
> 0.05	31.9 (606)	*NA*	*NA*
Area-level socioeconomic indicator at pregnancy			*NA*
Low level of deprivation	25.1 (466)	60.4 (4613)	*NA*
Medium-low level of deprivation	20.4 (379)	10.1 (770)	*NA*
Medium level of deprivation	15.9 (295)	10.9 (832)	*NA*
Medium-high level of deprivation	19.6 (364)	9.9 (757)	*NA*
High level of deprivation	19 (353)	8.8 (672)	*NA*
**Psychosocial factors**			
Psychiatric disorders during pregnancy	5.7 (111)	10.6 (689)	20.4 (185) ^g^
Free/subsidised health insurance for very low income families or no complementary insurance	9.3 (179)	*NA*	55.4 (515)
**Access to health care**		*NA*	
Antenatal visits			
< 7	12.5 (234)		26.4 (134) ^h^
≥ 7	87.5 (1645)		73.6 (374) ^i^
**Antenatal preparation for parenting**			
No	45 (845)		*NA*
Yes all	36.2 (680)		*NA*
Yes some	18.8 (354)		*NA*

*Data characteristics before imputation, based on the selection of the population with available data on parental lifestyle pattern. A description of all variables is included in Supplementary Tables 2, and number of missing values in Supplementary Tables 3–5

**Maternal birth outside Ireland was an exclusion criterion in Lifeways

^A^ Value for category (< 1,200 or 1,200 €) ^b^ Value for category (1,201-2,200 €), ^c^ Value for category (> 2,200 €)

^D^ < 600£/week ^e^ ≥ 600£/week. ^f^ Facility richness not categorised in Generation R

^g^ considered moderately or extremely anxious or depressed ^h^ < 6 antenatal visits ^i^ ≥6 antenatal visits

The results of the hierarchical multivariable linear regressions of parental lifestyle patterns are presented in Tables 2, 3 and 4.

**Table 2:** Imputed hierarchical multivariable linear regression analyses with parental lifestyle patterns. The EDEN study (*N* = 1962)

Parental lifestyle pattern 1: high parental smoking, poor-quality maternal diet and low leisure PA						
	Model 1		Model 2		Model 3	
	β (95% CI)	P-value	β (95% CI)	P-value	β (95% CI)	P-value
**Socioeconomic and demographic characteristics**						
**Centre**						
Poitiers	ref	ref				
Nancy	0.04 (-0.08, 0.15)	0.53	0.04 (-0.09, 0.16)	0.55	0.07 (-0.06, 0.20)	0.28
**Maternal education level**						
High	ref	ref				
Medium	**0.35 (0.21, 0.50)**	**< .001**	0.35 (0.21, 0.50)	< .001	0.32 (0.18, 0.47)	< .001
Low	**0.93 (0.67, 1.18)**	**< .001**	0.92 (0.67, 1.18)	< .001	0.83 (0.57, 1.09)	< .001
**Paternal education**						
High	ref	ref				
Medium	**0.31 (0.17, 0.45)**	**< .001**	0.30 (0.16, 0.45)	< .001	0.29 (0.14, 0.43)	< .001
Low	**0.51 (0.29, 0.73)**	**< .001**	0.50 (0.28, 0.73)	< .001	0.47 (0.24, 0.69)	< .001
**Maternal employment**						
Employed/self-employed	ref	ref				
Not employed	**0.28 (0.13, 0.44)**	**< .001**	0.27 (0.11, 0.42)	< .001	0.24 (0.08, 0.40)	0.003
**Paternal employment**						
Employed/self-employed	ref	ref				
Not employed	0.08 (-0.14, 0.30)	0.46	0.08 (-0.14, 0.30)	0.47	0.04 (-0.18, 0.26)	0.72
**Parity**						
Primiparous	ref	ref				
Multiparous	**0.16 (0.04, 0.29)**	**0.01**	0.16 (0.04, 0.29)	0.01	0.03 (-0.12, 0.17)	0.71
**Household income**						
4th quartile (highest)	ref	ref				
3rd quartile	0.12 (-0.04, 0.29)	0.14	0.13 (-0.04, 0.29)	0.13	0.13 (-0.03, 0.30)	0.11
2nd quartile	**0.20 (0.02, 0.38)**	**0.03**	0.20 (0.02, 0.38)	0.03	0.20 (0.02, 0.38)	0.03
1st quartile	**0.41 (0.16, 0.67)**	**0.001**	0.41 (0.16, 0.67)	0.002	0.35 (0.09, 0.60)	0.009
**Parents live together**						
Yes	ref	ref				
No	-0.09 (-0.36, 0.18)	0.52	-0.10 (-0.37, 0.17)	0.47	-0.13 (-0.40, 0.15)	0.37
**Maternal age**	**-0.05 (-0.06, -0.03)**	**< .001**	-0.05 (-0.06, -0.03)	< .001	− 0.04 (-0.06, -0.03)	< .001
**Paternal age**	-0.01 (-0.02, 0.01)	0.22	-0.01 (-0.02, 0.01)	0.22	-0.01 (-0.02, 0.00)	0.18
**Mother born abroad**						
No	ref	ref				
Yes	**-0.39 (-0.70, -0.09)**	**0.01**	-0.39 (-0.70, -0.09)	0.01	-0.41 (-0.71, -0.10)	0.01
**Father born abroad**						
No	ref	ref				
Yes	-0.22 (-0.46, 0.03)	0.08	-0.23 (-0.48, 0.02)	0.07	-0.26 (-0.51, -0.01)	0.038
**Urban environment**						
**Access to green space**			0.06 (-0.01, 0.14)	0.10	0.06 (-0.01, 0.14)	0.11
**Population density**			-0.07 (-0.16, 0.02)	0.14	-0.06 (-0.15, 0.03)	0.17
**Road and rail traffic**			0.00 (-0.11, 0.11)	0.99	-0.01 (-0.12, 0.10)	0.9
**Street connectivity density**			0.01 (-0.09, 0.10)	0.89	0.01 (-0.09, 0.11)	0.84
**Food facility density**						
Slightly unhealthy environment			ref	ref		
Highly unhealthy environment			0.07 (-0.10, 0.24)	0.40	0.07 (-0.09, 0.24)	0.38
**Facility richness**						
0			-0.02 (-0.24, 0.20)	0.88	-0.02 (-0.24, 0.20)	0.87
0-0.05			-0.03 (-0.21, 0.16)	0.79	-0.02 (-0.21, 0.16)	0.79
> 0.05			ref	ref		
**Area-level SES indicator (deprivation index in quintiles) during pregnancy**						
Low level of deprivation			ref	ref		
Medium-low level of deprivation			0.06 (-0.11, 0.24)	0.47	0.06 (-0.11, 0.23)	0.48
Medium level of deprivation			0.14 (-0.05, 0.33)	0.14	0.15 (-0.04, 0.34)	0.12
Medium-high level of deprivation			0.09 (-0.11, 0.28)	0.38	0.07 (-0.12, 0.26)	0.47
High level of deprivation			0.12 (-0.08, 0.32)	0.25	0.10 (-0.10, 0.30)	0.34
**Psychosocial factors and health-care access**						
**Psychiatric disorders during pregnancy**						
No					ref	ref
Yes					0.00 (-0.25, 0.25)	1
**Free/subsidised health insurance for very low-income families or no complementary insurance**						
No					ref	ref
Yes					0.22 (-0.01, 0.45)	0.06
**Antenatal visits**						
**< 7**					0.06 (-0.12, 0.24)	0.49
**≥ 7**					ref	ref
**Antenatal preparation for parenting**						
No					**0.29 (0.14, 0.44)**	**< .001**
Yes for all					ref	ref
Yes for some					0.13 (-0.03, 0.30)	0.12
**Parental lifestyle pattern 2: low parental BMI and high GWG**						
	**Model 1**		**Model 2**		**Model 3**	
	**β (95% CI)**	**P-value**	**β (95% CI)**	**P-value**	**β (95% CI)**	**P-value**
**Socioeconomic and demographic characteristics**						
**Centre**						
Poitiers	ref	ref	ref	ref		
Nancy	0.06 (-0.05, 0.16)	0.28	0.01 (-0.10, 0.12)	0.84	0.04 (-0.07, 0.16)	0.45
**Maternal education level**						
High	ref	ref	ref	ref		
Medium	-0.04 (-0.17, 0.09)	0.51	-0.03 (-0.16, 0.10)	0.65	-0.02 (-0.15, 0.11)	0.73
Low	0.15 (-0.08, 0.37)	0.21	0.15 (-0.08, 0.38)	0.19	0.18 (-0.05, 0.41)	0.13
**Paternal education**						
High	ref	ref	ref	ref		
Medium	**-0.25 (-0.38, -0.13)**	**< .001**	-0.23 (-0.36, -0.10)	< .001	-0.22 (-0.35, -0.10)	< .001
Low	-0.14 (-0.33, 0.06)	0.18	-0.11 (-0.31, 0.09)	0.27	-0.10 (-0.30, 0.10)	0.34
**Maternal employment**						
Employed/self-employed	ref	ref				
Not employed	-0.03 (-0.17, 0.11)	0.65	-0.03 (-0.17, 0.11)	0.69	-0.02 (-0.16, 0.12)	0.74
**Paternal employment**						
Employed/self-employed	ref	ref	ref	ref		
Not employed	0.03 (-0.16, 0.22)	0.76	0.01 (-0.18, 0.20)	0.93	0.03 (-0.16, 0.22)	0.75
**Parity**						
Primiparous	ref	ref	ref	ref		
Multiparous	**-0.22 (-0.33, -0.10)**	**< .001**	-0.20 (-0.32, -0.09)	< .001	-0.17 (-0.30, -0.04)	0.009
**Household income**						
4th quartile (highest)	ref	ref				
3rd quartile	**-0.19 (-0.33, -0.04)**	**0.01**	-0.18 (-0.33, -0.03)	0.02	-0.18 (-0.33, -0.03)	0.02
2nd quartile	**-0.32 (-0.48, -0.16)**	**< .001**	-0.32 (-0.48, -0.16)	< .001	-0.32 (-0.48, -0.16)	< .001
1st quartile	**-0.26 (-0.48, -0.03)**	**0.03**	-0.25 (-0.48, -0.02)	0.03	-0.24 (-0.47, -0.01)	0.04
**Parents live together**						
Yes	ref	ref				
No	0.02 (-0.22, 0.26)	0.9	0.03 (-0.21, 0.27)	0.8	0.06 (-0.18, 0.30)	0.62
**Maternal age**	-0.01 (-0.03, 0.00)	0.11	-0.01 (-0.03, 0.00)	0.11	-0.01 (-0.03, 0.00)	0.12
**Paternal age**	**-0.02 (-0.04, -0.01)**	**< .001**	-0.02 (-0.03, -0.01)	0.001	-0.02 (-0.03, -0.01)	0.002
**Mother born abroad**						
No	ref	ref				
Yes	-0.23 (-0.50, 0.04)	0.10	-0.26 (-0.54, 0.01)	0.06	-0.30 (-0.57, -0.02)	0.03
**Father born abroad**						
No	ref	ref				
Yes	0.02 (-0.20, 0.23)	0.89	0.00 (-0.21, 0.22)	0.99	0.03 (-0.19, 0.25)	0.79
**Urban environment**						
**Access to green space**			-0.04 (-0.10, 0.02)	0.17	-0.04 (-0.10, 0.02)	0.18
**Population density**			**0.13 (0.06, 0.21)**	**< .001**	0.13 (0.06, 0.21)	< .001
**Road and rail traffic**			0.00 (-0.09, 0.08)	0.98	0.00 (-0.09, 0.08)	0.96
**Street connectivity density**			0.00 (-0.09, 0.08)	0.95	0.00 (-0.08, 0.08)	1
**Food facility density**						
Slightly unhealthy environment			ref	ref		
Highly unhealthy environment			0.04 (-0.11, 0.19)	0.61	0.04 (-0.11, 0.19)	0.58
**Facility richness**						
0			0.00 (-0.20, 0.19)	0.97	0.01 (-0.19, 0.21)	0.91
0-0.05			-0.05 (-0.21, 0.12)	0.58	-0.04 (-0.20, 0.13)	0.67
> 0.05			ref	ref		
**Area-level socioeconomic indicator during pregnancy**						
Low level of deprivation			ref	ref		
Medium-low level of deprivation			-0.02 (-0.17, 0.13)	0.81	-0.02 (-0.17, 0.13)	0.81
Medium level of deprivation			0.00 (-0.16, 0.17)	0.96	0.01 (-0.16, 0.17)	0.95
Medium-high level of deprivation			-0.11 (-0.28, 0.06)	0.21	-0.10 (-0.27, 0.07)	0.23
High level of deprivation			**-0.21 (-0.39, -0.03)**	**0.02**	-0.20 (-0.38, -0.02)	0.03
**Psychosocial factors and health-care access**						
**Psychiatric disorders during pregnancy**						
No					ref	ref
Yes					**-0.29 (-0.51, -0.07)**	**0.01**
**Free/subsidised health insurance for very low-income families or no complementary insurance**						
No					ref	ref
Yes					-0.07 (-0.27, 0.14)	0.53
**Antenatal visits**						
**< 7**					**0.18 (0.02, 0.34)**	**0.03**
**≥ 7**					ref	ref
**Antenatal preparation for parenting**						
No					-0.09 (-0.23, 0.05)	0.19
Yes for all					ref	ref
Yes for some					-0.11 (-0.25, 0.04)	0.15

PA: physical activity; model 1 included socioeconomic, and demographic factors, model 2 included urban environment factors and model 3 psychosocial factors and access to health care. For the sake of parsimony, the effect of each variable was adjusted for the other variables from the same block, and additionally adjusted for variables from the preceding block. Coefficients are interpreted when the variable appear the first time but we showed effects for further models for indication

**Table 3:** Imputed hierarchical multivariable linear regression analyses with parental lifestyle patterns. Generation R study. (*N* = 8765)

Parental lifestyle pattern 1: high parental smoking, poor-quality maternal diet						
	Model 1		Model 2		Model 3	
	β (95% CI)	P-value	β (95% CI)	P-value	β (95% CI)	P-value
**Socioeconomic and demographic characteristics**						
**Maternal education level**						
High	ref	ref	ref	ref	ref	ref
Medium	**0.44 (0.37, 0.51)**	**< .001**	0.43 (0.36, 0.51)	< .001	0.43 (0.36, 0.50)	< .001
Low	**0.47 (0.35, 0.58)**	**< .001**	0.45 (0.34, 0.57)	< .001	0.45 (0.34, 0.57)	< .001
**Paternal education**						
High	ref	ref	ref	ref	ref	ref
Medium	**0.37 (0.30, 0.45)**	**< .001**	0.37 (0.30, 0.45)	< .001	0.37 (0.30, 0.45)	< .001
Low	**0.63 (0.49, 0.76)**	**< .001**	0.62 (0.49, 0.76)	< .001	0.62 (0.48, 0.76)	< .001
**Maternal employment**						
Employed/self-employed	ref	ref	ref	ref	ref	ref
No employed	**0.16 (0.08, 0.24)**	**< .001**	0.16 (0.08, 0.24)	< .001	0.15 (0.07, 0.23)	< .001
**Paternal employment**						
Employed/self-employed	ref	ref	ref	ref	ref	ref
Not employed	-0.05 (-0.21, 0.11)	0.53	-0.06 (-0.21, 0.10)	0.49	-0.06 (-0.21, 0.10)	0.48
**Parity**						
Primiparous	ref	ref	ref	ref	ref	ref
Multiparous	**0.14 (0.08, 0.20)**	**< .001**	0.15 (0.09, 0.21)	< .001	0.15 (0.09, 0.21)	< .001
**Household income**						
4th quartile (highest) > 2200	ref	ref	ref	ref	ref	ref
3rd quartile (1200–2200)	**0.16 (0.09, 0.24)**	**< .001**	0.15 (0.08, 0.23)	< .001	0.15 (0.08, 0.23)	< .001
2nd quartile < = 1200	**0.19 (0.08, 0.30)**	**0.001**	0.18 (0.07, 0.30)	0.0015	0.17 (0.06, 0.29)	0.003
1st quartile						
**Parents live together**						
Yes	ref	ref	ref	ref	ref	ref
No	**0.24 (0.15, 0.33)**	**< .001**	0.24 (0.14, 0.33)	< .001	0.23 (0.14, 0.32)	< .001
**Maternal age**	**-0.01 (-0.02, -0.01)**	**< .001**	-0.01 (-0.02, -0.01)	< .001	-0.01 (-0.02, -0.01)	< .001
**Paternal age**	**-0.01 (-0.02, -0.01)**	**< .001**	-0.01 (-0.02, 0.00)	< .001	-0.01 (-0.02, 0.00)	< .001
**Mother born abroad**						
No	ref	ref	ref	ref	ref	ref
Yes	**-0.32 (-0.39, -0.25)**	**< .001**	-0.33 (-0.40, -0.26)	< .001	-0.33 (-0.41, -0.26)	< .001
**Father born abroad**						
No	ref	ref	ref	ref	ref	ref
Yes	**-0.17 (-0.24, -0.09)**	**< .001**	-0.18 (-0.26, -0.10)	< .001	-0.19 (-0.27, -0.11)	< .001
**Population density**			0.01 (-0.02, 0.04)	0.38	0.01 (-0.02, 0.04)	0.37
**Access to green space**			0.00 (-0.03, 0.03)	0.96	0.00 (-0.03, 0.03)	0.96
**Road and rail traffic**			-0.01 (-0.04, 0.02)	0.49	-0.01 (-0.04, 0.02)	0.48
**Street connectivity density**			0.00 (-0.04, 0.04)	0.89	0.00 (-0.04, 0.04)	0.91
**Food facility density**						
Slightly unhealthy environment			ref	ref	ref	ref
Highly unhealthy environment			0.00 (-0.10, 0.09)	0.93	-0.01 (-0.10, 0.09)	0.91
**Facility richness**			0.01 (-0.03, 0.05)	0.65	0.01 (-0.03, 0.05)	0.63
**Urban environment**						
**Area-level socioeconomic indicator during pregnancy**						
Low level of deprivation			ref	ref	ref	ref
Medium-low level of deprivation			-0.08 (-0.22, 0.07)	0.32	-0.07 (-0.22, 0.07)	0.33
Medium level of deprivation			0.00 (-0.14, 0.13)	0.96	0.00 (-0.14, 0.13)	0.97
Medium-high level of deprivation			0.00 (-0.15, 0.15)	0.99	0.00 (-0.15, 0.15)	1
High level of deprivation			0.06 (-0.07, 0.18)	0.39	0.06 (-0.07, 0.18)	0.39
**Psychosocial factors**						
**Psychiatric disorders during pregnancy**						
No					ref	ref
Yes					**0.13 (0.02, 0.24)**	**0.02**
**Parental lifestyle pattern 2: low parental BMI and high GWG**						
	**Model 1**		**Model 2**		**Model 3**	
	**β(95% CI)**	**P-value**	**β (95% CI)**	**P-value**	**β (95% CI)**	**P-value**
**Maternal education level**						
High	ref	ref	ref	ref	ref	ref
Medium	**-0.20 (-0.27, -0.14)**	**< .001**	-0.19 (-0.26, -0.13)	< .001	-0.19 (-0.26, -0.13)	< .001
Low	**-0.23 (-0.33, -0.13)**	**< .001**	-0.23 (-0.33, -0.13)	< .001	-0.23 (-0.33, -0.13)	< .001
**Paternal education**						
High	ref	ref	ref	ref	ref	ref
Medium	**-0.07 (-0.14, -0.01)**	**0.03**	-0.06 (-0.13, 0.00)	0.06	-0.06 (-0.13, 0.00)	0.05
Low	-0.08 (-0.18, 0.03)	0.17	-0.07 (-0.18, 0.04)	0.22	-0.07 (-0.18, 0.04)	0.2
**Maternal employment**						
Employed/self-employed	ref	ref	ref	ref	ref	ref
No employed	-0.01 (-0.08, 0.07)	0.87	-0.01 (-0.08, 0.06)	0.78	-0.01 (-0.09, 0.06)	0.71
**Paternal employment**						
Employed/self-employed	ref	ref	ref	ref	ref	ref
Not employed	**-0.11 (-0.21, -0.01)**	**0.03**	-0.11 (-0.21, -0.02)	0.03	-0.11 (-0.21, -0.02)	0.025
**Parity**						
Primiparous	ref	ref	ref	ref	ref	ref
Multiparous	**-0.28 (-0.33, -0.23)**	**< .001**	-0.26 (-0.32, -0.21)	< .001	-0.26 (-0.32, -0.21)	< .001
**Household income**						
4th quartile (highest) > 2,200	ref	ref	ref	ref	ref	ref
3rd quartile (1,200-2,200)	0.02 (-0.05, 0.09)	0.67	0.01 (-0.06, 0.08)	0.77	0.01 (-0.06, 0.08)	0.79
2nd quartile < = 1,200	0.03 (-0.07, 0.13)	0.52	0.03 (-0.07, 0.13)	0.50	0.03 (-0.07, 0.13)	0.55
1st quartile						
**Parents live together**						
Yes	ref	ref	ref	ref	ref	ref
No	**0.20 (0.12, 0.28)**	**< .001**	0.20 (0.12, 0.28)	< .001	0.20 (0.12, 0.28)	< .001
**Maternal age**	**0.01 (0.00, 0.01)**	**0.04**	0.01 (0.00, 0.01)	0,035	0.01 (0.00, 0.01)	0.03
**Paternal age**	**-0.01 (-0.02, 0.00)**	**< .001**	-0.01 (-0.02, 0.00)	< .001	-0.01 (-0.02, 0.00)	< .001
**Mother born abroad**						
No	ref	ref	ref	ref	ref	ref
**Yes**	**-0.11 (-0.17, -0.05)**	**< .001**	-0.12 (-0.18, -0.06)	< .001	-0.12 (-0.18, -0.06)	< .001
**Father born abroad**						
No	ref	ref	ref	ref	ref	ref
Yes	**-0.22 (-0.28, -0.15)**	**< .001**	-0.22 (-0.29, -0.16)	< .001	-0.23 (-0.29, -0.16)	< .001
**Urban environment**						
**Population density**			-0.02 (-0.04, 0.01)	0.21	-0.02 (-0.04, 0.01)	0.21
**Access to green space**			-0.01 (-0.04, 0.01)	0.34	-0.01 (-0.04, 0.01)	0.33
**Road and rail traffic**			0.01 (-0.02, 0.04)	0.53	0.01 (-0.02, 0.04)	0.53
**Street connectivity density**			**0.04 (0.00, 0.07)**	**0.03**	0.04 (0.00, 0.07)	0.03
**Food facility density**						
Slightly unhealthy environment			ref	ref	ref	ref
High unhealthy environment			0.03 (-0.05, 0.12)	0.43	0.03 (-0.05, 0.12)	0.44
**Facility richness**			**0.04 (0.01, 0.07)**	**0.02**	0.04 (0.01, 0.07)	0.02
**Area-level SES indicator (deprivation index in quintiles) during pregnancy**						
Low level of deprivation			ref	ref	ref	ref
Medium-low level of deprivation			0.03 (-0.10, 0.16)	0.63	0.03 (-0.10, 0.16)	0.63
Medium level of deprivation			-0.04 (-0.16, 0.07)	0.44	-0.04 (-0.16, 0.07)	0.44
Medium-high level of deprivation			-0.03 (-0.16, 0.10)	0.66	-0.03 (-0.16, 0.10)	0.67
High level of deprivation			-0.05 (-0.17, 0.07)	0.41	-0.05 (-0.17, 0.07)	0.41
**Psychosocial factors**						
**Psychiatric disorders during pregnancy**						
No					ref	ref
Yes					0.05 (-0.05, 0.15)	0.3

Model 1 included socioeconomic, and demographic factors, model 2 included urban environment factors and model 3 psychosocial factors and access to health care. For the sake of parsimony, the effect of each variable was adjusted for the other variables from the same block, and additionally adjusted for variables from the preceding block. Coefficients are interpreted when the variable appear the first time but we showed effects for further models for indication

**Table 4:** Imputed hierarchical multivariable linear regression analyses with parental lifestyle patterns. The lifeways study. (*N* = 932)

Parental lifestyle pattern 1: High parental smoking, inflammatory diet, low maternal DASH and rather low paternal PA				
	Model 1		Model 3	
	β (95% CI)	P-value	β (95% CI)	P-value
**Socioeconomic and demographic characteristics**				
**Maternal education level**				
High	ref	ref	ref	ref
Medium	**0.45 (0.23, 0.67)**	**< .001**	0.37 (0.15, 0.59)	< .001
Low	**0.57 (0.30, 0.84)**	**< .001**	0.46 (0.19, 0.73)	< .001
**Paternal education**				
High	ref	ref	ref	ref
Medium	**0.35 (0.12, 0.59)**	**0.004**	0.29 (0.05, 0.53)	0.02
Low	**0.40 (0.16, 0.63)**	**< .001**	0.27 (0.04, 0.51)	0.02
**Maternal employment**				
Employed/self-employed	ref	ref	ref	ref
Not employed	-0.06 (-0.27, 0.15)	0.58	-0.13 (-0.34, 0.08)	0.22
**Paternal employment**				
Employed/self-employed	ref	ref	ref	ref
Not employed	0.33 (-0.46, 1.12)	0.42	0.32 (-0.44, 1.08)	0.41
**Parity**				
Primiparous	ref	ref	ref	ref
Multiparous	**0.31 (0.10, 0.52)**	**0.004**	0.32 (0.11, 0.54)	0.003
**Household income**				
4th quartile (highest) ≥ 600£/week	ref	ref	ref	ref
3rd quartile	NA	NA	NA	NA
2nd quartile	NA	NA	NA	NA
1st quartile (lowest) < 600£/week	**0.26 (0.05, 0.46)**	**0.01**	0.18 (-0.03, 0.38)	0.09
**Parents live together**				
Yes	ref	ref	ref	ref
No	0.21 (-0.02, 0.43)	0.07	0.16 (-0.06, 0.39)	0.16
**Maternal age**	**-0.06 (-0.09, -0.03)**	**< .001**	-0.05 (-0.08, -0.02)	< .001
**Paternal age**	-0.02 (-0.04, 0.01)	0.16	-0.02 (-0.04, 0.01)	0.20
**Psychosocial factors and health-care access**				
**Psychiatric disorders during pregnancy**				
No			ref	ref
Yes			0.18 (-0.03, 0.40)	0.10
**Free/subsidised health insurance for very low-income families or no complementary insurance**				
No			ref	ref
Yes			**0.49 (0.28, 0.70)**	**< .001**
**Antenatal visits**				
< 6 antenatal visits			0.14 (-0.14, 0.42)	0.32
≥ 6 antenatal visits			ref	ref

PA: physical activity. Model 1 included socio-economic and demographic factors, variable for model 2 are not available for Lifeways, and model 3 psychosocial factors and access to health care. For the sake of parsimony, the effect of each variable was adjusted for the other variables from the same block, and additionally adjusted for variables from the preceding block. Coefficients are interpreted when the variable appear the first time but we showed effects for further models for indication. Parent’s birthplace was not considered (maternal birth in Ireland was an inclusion criterion for Lifeways)

### Socioeconomic and sociodemographic characteristics

In the EDEN cohort, families with lower parental SEP (education level, household income, and employment) had higher scores on the pattern “high parental smoking, poor-quality maternal diet, and low leisure PA”, whereas older, foreign-born, and first-time mothers had lower scores on this pattern (Table 2). Consistent findings were observed in Generation R for the “high parental smoking and poor-quality maternal diet” lifestyle pattern. Moreover, mothers who lived alone scored higher on this pattern, whereas foreign-born or older fathers followed it less often (Table 3). Similarly, in Lifeways, parents whose education level or household income was low (< 600 £/week), and younger or multiparous mothers had higher scores on the “high parental smoking, inflammatory diet, low maternal DASH, and rather low paternal PA” pattern. No association with any other socioeconomic factor was observed (Table 4).

Parents with a low income, multiparous women, older fathers, and those with intermediate education levels scored lower on the “low parental BMI and high GWG” pattern in EDEN (Table 2) and Generation R (Table 3). In Generation R, lower scores on this pattern were observed among women with low education levels, unemployed fathers, and parents born abroad; conversely, older women and those living alone followed this pattern more closely (Table 3).

### Urban environment

Urban environmental factors were not associated with the first lifestyle pattern in either EDEN or Generation R. However, population density (in EDEN), street connectivity density and facility richness (in Generation R) were associated with the “low parental BMI and high GWG” pattern. Conversely, EDEN parents living in highly deprived areas had lower scores on this pattern (Tables 2 and 3).

### Psychosocial factors and access to health care

Mothers with psychological disorders during pregnancy scored higher on the “high parental smoking and poor-quality maternal diet” pattern in Generation R and lower on the “low parental BMI and high GWG” pattern in EDEN. When mothers did not attend any parenting preparation sessions during pregnancy and when households had either insurance for very low-income families or no private insurance, the parents had higher scores on the “high parental smoking, poor-quality maternal diet, and low leisure PA” lifestyle pattern in EDEN and on the “high parental smoking, inflammatory diet, low maternal DASH, and rather low paternal PA” pattern in Lifeways. Families who did not attend the recommended number of antenatal visits had higher scores on the “low parental BMI and high GWG” pattern in EDEN.

Parental and maternal lifestyle patterns showed consistent associations (Supplementary Tables 6–8). Sensitivity analysis yielded consistent findings (results not shown, but available on request).

## Discussion

### Summary of results

This study provides comprehensive insights into the socioecological correlates of parental lifestyle patterns in pregnancy, at multiple levels of influence, beyond individual SEP. We report consistent findings between countries in this large collaborative project with, where possible, harmonised data on participants from three European cohorts. Briefly, older parents, those born abroad, those with higher SEP or living in a more advantaged physical environment had healthier lifestyle patterns in pregnancy. Conversely, multiparous mothers or those who had developed psychiatric disorders during pregnancy had suboptimal lifestyles. Beyond individual factors, we found positive associations between population density, street connectivity density, and facility richness in the immediate neighbourhood, and the second pattern “low parental BMI and high GWG”. Conversely parents living in highly deprived areas had lower scores on this pattern. Finally, positive associations were observed between factors related to optimal health care, such as adequate health insurance coverage and parenting preparation sessions, and adherence to healthier combinations of behaviours.

### Interpretation

Strong socioeconomic inequalities in health exist from early childhood [32]. For example, children born to parents with lower SEP are more likely to develop OW/OB in early life [33]. The pathways by which parental SEP affects children’s health are complex and include parents’ health behaviours as early as the preconception period [12]. Our results reflect previously reported evidence of an inverse relation between SEP and antenatal risk factors for childhood obesity, such as maternal prepregnancy BMI, poor-quality diet, and smoking during pregnancy [12].

Parents born abroad demonstrated lower adherence to the “high parental smoking and poor-quality maternal diet” pattern. This is consistent with previous findings from the French national ELFE birth cohort [27] that immigrant mothers and those descending from immigrants had healthier diets and ate less processed food, consistent with reports that the acculturation process leads women toward a westernised diet [27]. Immigrant women in the ELFE cohort also smoked less in the perinatal period than non-immigrants, but were at higher risk of developing overweight or obesity, especially those born in sub-Saharan Africa [34].

Multiparity was consistently associated with a suboptimal lifestyle during pregnancy in all three cohorts. The greater economic and time constraints due to the siblings’ presence likely explains this finding. Time and money facilitate parental engagement in healthy lifestyles [35].

Over the last decade, there has been a notable scientific and political interest in understanding the social determinants of health. A growing consensus holds that a broader social environment and structural drivers significantly shape overall health status. These drivers are also called the “upstream” factors, differentiated from the downstream influences related to individual traits [36]. Research, predominantly focused on social factors that determine life-course health, has paid less attention to the influence of the built environment in early life [37]. A scoping review addressing this subject in relation to adult PA, dietary behaviours, and obesity reports that increased access to grocery stores and farmers’ markets is positively associated with dietary quality [26]. The authors also conclude that a healthier overall food environment, greater access to parks and playgrounds, as well as supermarkets, are associated with a healthier weight, along with high population density. This is consistent with the positive association we observed between the “low parental BMI and high GWG” pattern and population density, facility richness, and street connectivity density. These findings support the view that a well-designed urban environment may influence parents’ and children’s lifestyle favourably. Complementary to the structural levers/barriers of the built environment, other social determinants such as community norms, networks, social support, and interpretation of families’ perception of the environment should be considered [38]. They are, however, beyond the scope of this study.

Antenatal care consists in a series of recommended clinical visits during pregnancy and the option to attend parenting preparation sessions designed to promote both the parents’ and child’s health and well-being. Our results showed that families who followed both recommendations and those with complementary health insurance were more likely to adhere to healthier lifestyles. We did not obtain consistent associations across cohorts, perhaps because these health promotion programmes, providing advice on diverse topics (e.g. diet, PA, smoking, chemical exposures, breastfeeding), are country-specific. As the EDEN study was designed in the 2000s, we can suspect that these associations would be stronger today, with greater communication on the importance of parenting preparation and greater inclusion of fathers, who are more involved in child care [39].

We remain cautious about the interpretation of the model including block 3 factors. While better access to health care might well be associated with more favourable health behaviours, reverse causality cannot be excluded, i.e., parents who are more health-conscious may be more likely to follow recommendations during pregnancy. Finally, other studies have shown positive relations between an unhealthy overall lifestyle (smoking, low level of PA…) and the risk of perinatal depression and anxiety during pregnancy [28, 40, 41]. These bi-directional associations between lifestyle and mental health, as well as the influence of external factors such as social support, remain relatively understudied.

### Public health perspectives

Interventions aimed at influencing health behaviours have typically concentrated on individual capacity to change. They are increasingly criticised for over-emphasising individual choice and personal responsibility, without considering the structural barriers the targeted populations may face, especially in socially disadvantaged settings [36]. Our findings show that unemployment (entailing a smaller social network and lower self-esteem), low income (budgetary constraints and trade-offs), a low education level (suboptimal knowledge and health literacy), disadvantaged urban environment (limited proximity to the healthy food sources and services available through facility richness) all represent barriers to engagement in a healthier lifestyle. Having a socioecological perspective means recognising that structural facilitators (e.g., employment, culturally appropriate information, greater income, enhanced availability and access to services) must be mobilised in multi-level interventions to empower people to hear, understand, and adhere to public health recommendations [36].

The first 1000 days of life represent an important period when parents are encouraged — and more likely — to change their own behaviours to optimise their future child’s health. A recent systematic review has evaluated the effectiveness of interventions during the first 1000 days in improving lifestyle behaviours and preventing OW/OB in children from socioeconomically disadvantaged families [35]. None of the programs reviewed included or evaluated structural components such as incentives to facilitate access to healthy foods, social and health support services, or the potential role of urban design in facilitating changes in family lifestyles [35]. As Francis-Oliviero et al. argue, Marmot’s theory of proportionate universalism is a useful perspective to apply to “reduce the social gradient in health, by providing universal access to health services, but with a scale and intensity that is proportionate to the level of disadvantage” [42, 43]. In addition to the needed structural changes to the upstream drivers of health inequalities, health-care professionals and social-service providers must play a vital role in supporting these families and encouraging them to adopt a healthier lifestyle by co-designing and adapting prevention measures to the family and social context.

### Strengths and limitations

The novelty of our study is the socioecological framework used to encompass several dimensions of SEP and other social determinants, in conjunction with an integrative approach to the various health behaviours. Although the design of our socioecological model did not allow us to determine causal pathways, the hierarchical approach prevented overadjustment for mediating variables that might underestimate associations between distal factors and the outcome. We aimed to reduce heterogeneity by harmonising data across studies, through similar definitions and similar categorisation of variables. The use of multiple imputation techniques limited selection bias due to missing data in the socioecological model variables. However, we cannot rule out measurement errors and information bias, given that most data are self-reported. Additionally, we cannot exclude residual confounding with unmeasured factors that might influence parental lifestyles (social support, work conditions, follow-up with specialists during pregnancy). It is worth noting that we found similar associations between countries despite contexts that differed in their proportions of mothers born abroad, types of urban infrastructure, and health-care access — all points that reinforce the robustness of our conclusions. However, since the data were collected two decades ago, it is possible that the strength of the associations under study may differ today due to the increasingly obesogenic environment and changes in health care access.

## Conclusion

Obtained by an integrative approach to assess lifestyle patterns among mother-father pairs, these results confirm the importance of a higher SEP to facilitate optimal behaviours and BMI status during pregnancy. We further highlighted the role of structural factors such as urban environment and health-care access, with great consistency between European cohorts. These findings underline the need to consider not only individual characteristics but also the living environment, to empower parents in improving their lifestyle. Further research efforts should focus more on understanding the mechanisms through which structural factors influence parents’ lifestyles and how to change them to reduce social inequalities in health within families more effectively.

## Electronic supplementary material

Below is the link to the electronic supplementary material.

Supplementary Material 1

Supplementary Material 2

## Data Availability

Some or all datasets generated during and/or analysed during the current study are not publicly available but are available from the corresponding author on reasonable request and signing of the appropriate data-sharing agreement, and approval by the steering committees of each cohort.

## Abbreviations

BMI: Body mass index
DASH: Dietary approach to stop hypertension
E-DII: Energy-adjusted dietary inflammatory index
GWG: Gestational weight gain
OW/OB: Overweight and obesity
PA: Physical activity
PCA: Principal component analyses
SEP: Socioeconomic position
