# Supplementary material for: Socioecological correlates of parental lifestyle patterns during the antenatal period
Source: Int J Behav Nutr Phys Act. 2025 Feb 13;22:18. doi: 10.1186/s12966-024-01697-1 (PMC11827224; doi:10.1186/s12966-024-01697-1)
Supplement: Supplementary file 2 — Supplementary Material 2 [file 12966_2024_1697_MOESM2_ESM.docx]

**Supplementary Material**

**Supplementary Table 1: Description of the three European cohorts involved and ethics statement.**

| **EDEN** | **Generation R** | **Lifeways** |
| --- | --- | --- |
| The EDEN birth cohort was designed to evaluate the early pre-, and post-natal determinants of child health and development. Study participation was proposed to all women visiting the antenatal clinic before 24 weeks’ gestation. Between 2003 and 2006, 2002 (53%) 18 to 45-year-old pregnant women were recruited in two centres: Nancy and Poitiers hospitals and 89.2% of fathers agreed to participate ^16^  The EDEN study received approval from the ethics committee of Kremlin Bicêtre Hospital (02–70) and the Commission Nationale de l’Informatique et des Libertés, the French data privacy authority | Generation R Study is a population-based prospective cohort study from fetal life until young adulthood in Rotterdam, the Netherlands.^18^ It includes 9778 mothers of whom 91% (n=8880) were recruited between early pregnancy and birth. Fathers were invited to participate and 71% (n = 6347) agreed. Parental data were collected repeatedly during pregnancy by physical examinations and questionnaires.  Study approval was obtained by the Medical Ethical Committee of the Erasmus Medical Center, University Medical Centre, Rotterdam (MEC 198.782/2001/31) | The Lifeways Cross-Generation Cohort Study is a cohort that aimed to document health status and lifestyle of the family members and establish patterns and links across generations.^15^ Mothers (n=1,124) were initially recruited by a midwife during their first antenatal visit in two maternity hospitals in the Republic of Ireland between 2001 and 2003; they gave birth to 1,094 live infants. The participating mothers' partners (biological fathers) were also directly contacted by the Lifeways research team at recruitment (participant mothers having given their contact details at their booking visit) and invited to participate (n=333 agreed). Longitudinal follow-up was conducted with linkage data to hospital and general practice records.  Ethical approval has been obtained for all stages of the Lifeways study from research ethics committees of the Coombe University Hospital, Dublin, University College Dublin, Irish College of General Practitioners, St Vincent’s University Hospital and University College Hospital, Galway, Ireland. |

**Supplementary Table 2: List of socio-ecological variables available in the three cohorts**

| **Variable** | **Description** | **Availability in each cohort (EDEN, GEN-R, Lifeways)** |
| --- | --- | --- |
| **Socioeconomic and demographic factors** |  |  |
| Mother born abroad | Whether mothers were born outside the cohort country  Yes / No (reference) | EDEN: evaluated at inclusion (24 weeks of gestation)  Gen-R: at enrolment  Not in Lifeways: maternal birth outside Ireland was an exclusion criterion |
| Father born abroad | Whether fathers were born outside the cohort country  Yes / No (reference) | EDEN: evaluated at inclusion (24 weeks of gestation)  Gen-R: at enrolment  Not collected in Lifeways |
| Maternal age | Continuous (in years) | Yes at delivery |
| Paternal age | Continuous (in years) | Yes at delivery |
| Parents live together | Cohabitation status of the mother (age ≥0 year and <1 year): does she live together with her partner? Yes (reference)/No | Yes  EDEN: combined information used at pregnancy, 4 and 8 months after birth  Gen-R: evaluated during the antenatal period  Lifeways: during the 1^st^ trimester of pregnancy |
| Maternal education | Level of education based on the highest on-going or completed education Classification according to International Standard Classification of Education 97/2011 (ISCED-97/2011) High (reference): Short cycle tertiary, Bachelor, Masters, Doctoral or equivalent (ISCED-2011: 5-8, ISCED-97: 5-6) Medium: Upper secondary, Post-secondary non-tertiary (ISCED-2011: 3-4, ISCED-97: 3-4) Low: No education; early childhood; pre-primary; primary; lower secondary or second stage of basic education. (ISCED-2011: 0-2, ISCED-97: 0-2) | Yes  EDEN: evaluated at inclusion (24 weeks of gestation)  Gen-R: evaluated during antenatal period  Lifeways: during the 1^st^ trimester of pregnancy |
| Paternal education | ISCED classification as above - High (reference), Medium, Low | Yes  Evaluated at inclusion (24 weeks of gestation) in EDEN  Gen-R: evaluated during the antenatal period  Lifeways: during the 1^st^ trimester of pregnancy |
| Maternal employment status | Occupational status of the mother Employed/self-employed includes maternity leave if she was employed before commencing maternity leave.  Use as Employed/self-employed (reference) vs no employed (Inactive/other (receiving benefits or pension etc.), Domestic tasks (housewife etc.), Student, apprentice, Unemployed). | Yes  Evaluated at inclusion (24 weeks of gestation) in EDEN  Gen-R: evaluated during antenatal period  Lifeways: during the 1^st^ trimester of pregnancy |
| Paternal employment status | Use as Employed/self-employed (reference) vs no employed | Yes  Evaluated at inclusion (24 weeks of gestation) in EDEN  Gen-R: evaluated during antenatal period  Lifeways: during the 1^st^ trimester of pregnancy |
| Parity | Use as primiparous (reference) vs multiparous | Yes |
| Household income | Total yearly income of the household | Yes  EDEN: combined information used at pregnancy and 12 months in EDEN  Total household income per unit of consumption categorized into quartiles in EDEN (low/medium-low/medium-high/high). This indicator is calculated by allocating different weights to each member of a household (1 consumption unit for the first adult in the household, 0.5 for other household members aged 14 or over, and 0.3 for children younger than 14 years) based on the French National Institute of Statistics and Economic Studies ^42^.  Gen-R: questionnaire during pregnancy. Household income was categorized as: ≤1200, 1201-2200, >2,200 €/month.  Lifeways: the household’s total net income per week (take-home family weekly income from all sources including social benefits) was categorized as <600 and ≥600 £/week. During the 1^st^ trimester of pregnancy |
| **Urban environment at pregnancy** |  |  |
| Population density | Population density, inhabitants per square kilometre | EDEN, Gen-R |
| Access to green space | Residential proximity to major green space (EU defines this as living within 300 m of public open area with more than 5000 m2) during pregnancy | EDEN, Gen-R |
| Road and rail traffic | Percentage of trans (road and rail network and associated land, fast transit roads and associated land, other roads and associated land, railways and associated land) land use within a buffer of 300 m at birth | EDEN, Gen-R |
| Street connectivity density | Intersection density was defined as the number of road intersections — that are not dead-ends — inside a buffer of 300 meters, divided by the area in square km of each buffer | EDEN, Gen-R |
| Food facility density | The unhealthy food environment variable equals the number of unhealthy facilities in the 300-m buffer divided by the area of the 300-m buffer | EDEN, Gen-R |
| Facility richness | Facilities were all points of interest for pedestrians as part of their daily life activities, such as restaurants, shops, medical centres, schools, libraries, etc. Facility richness index: equals the number of different facility types present divided by the maximum potential number of facility types specified, in a buffer of 300 meters. | EDEN, Gen-R |
| Area-level socioeconomic indicator (deprivation index in quintiles) during pregnancy | Levels of deprivation: high, medium-high, medium, medium-low, and low (reference) | EDEN: Use of the French European deprivation index. It includes Overcrowding, No access to a system of central or electric heating, Non-owner, Unemployment, Foreign nationality, No access to a car, Unskilled worker–farm worker, Household with more than six, Low level of education, Single-parent household.  Gen-R: Use of Status scores calculated by the social and cultural planning office of the Dutch Government. The status score indicates the social status of a neighbourhood compared to other neighbourhoods in the Netherlands. The social status of a neighbourhood is derived from a number of characteristics of the people who live there: their education, income, and position on the labour market |
| **Psychosocial factors and health-care access** |  |  |
| Maternal psychiatric disorders during pregnancy | Any psychiatric disorders (including anxiety, depression etc..) during pregnancy  Self-reported, specific questionnaires  Yes/ No (reference) | All cohorts  EDEN: Whether women have taken tranquilizers, antidepressants or anxiolytics during pregnancy (self-reported after birth)  Gen-R: Psychological symptoms during pregnancy were assessed using the Brief Symptom Inventory (53 items); the total psychopathology score was used to determine whether pregnant mothers had problems  Lifeways: Moderately or extremely anxious or depressed /vs not depressed (self-reported during the 1^st^ trimester of pregnancy) |
| Health insurance | Free health insurance for very low income families or no private insurance Yes/No (reference) | EDEN, Lifeways  EDEN: Free health insurance for very low income families or no complementary insurance/ vs private insurance (self-reported at inclusion)  Lifeways: General medical card or no private health insurance at baseline / vs private insurance (self-reported during the 1^st^ trimester of pregnancy) |
| Number of antenatal visits during pregnancy | The variable was categorised following the national recommendations and its distribution in two groups  Yes (reference)/No | EDEN, Lifeways  EDEN: <7 or ≥7 (self-reported after birth)  Lifeways: <6 or ≥6 (hospital information systems) |
| Parenting preparation sessions during pregnancy | - | EDEN  EDEN: none/some/all (reference) (self-reported after birth) |

**Supplementary Table 3: List of variables included for imputation and % of missing data in EDEN**

| Variable | Type of variable | Model used to predict missing data | Missing value % |
| --- | --- | --- | --- |
| Maternal age | Continuous | Predictive mean matching | 2.8 |
| Paternal age | Continuous | Predictive mean matching | 12.4 |
| Maternal employment | Binary | Logistic regression | 2.3 |
| Paternal employment | Binary | Logistic regression | 4.4 |
| Parity mother | Binary | Logistic regression | 3 |
| Maternal education | Categorical (3 categories) | Ordinal regression | 2.7 |
| Paternal education | Categorical (3 categories) | Ordinal regression | 2.6 |
| Maternal country of origin | Binary | Logistic regression | 2.9 |
| Paternal country of origin | Binary | Logistic regression | 3 |
| Cohabitation status | Binary | Logistic regression | 2.1 |
| Marital status | Binary | Logistic regression | 2.4 |
| Household income | Categorical (4 categories) | Ordinal regression | 3.2 |
| Road and rail traffic | Continuous | Predictive mean matching | 25.6 |
| Food facility density | Binary | Logistic regression | 3.3 |
| Facility richness | Categorical (3 categories) | Multinomial regression | 3.3 |
| Street connectivity density | Continuous | Predictive mean matching | 3.3 |
| Population density | Continuous | Predictive mean matching | 11.7 |
| Area level socioeconomic indicator | Categorical (4 categories) | Ordinal regression | 5.3 |
| Access to green space | Continuous | Predictive mean matching | 27.3 |
| Number of ultrasound scans | Continuous | Predictive mean matching | 3.9 |
| Number of antenatal visits | Binary | Logistic regression | 4.2 |
| Antenatal parenting preparation sessions | Categorical (3 categories) | Multinomial regression | 4.2 |
| Maternal health coverage | Binary | Logistic regression | 2.4 |
| Gestational age | Continuous | Predictive mean matching | 2.9 |
| Maternal prepregnancy BMI | Continuous | Predictive mean matching | 3.9 |
| Paternal BMI at inclusion | Continuous | Predictive mean matching | 9.1 |
| Gestational weight gain | Continuous | Predictive mean matching | 4.8 |
| Maternal smoking during pregnancy | Categorical (3 categories) | Ordinal regression | 17 |
| Paternal smoking during pregnancy | Categorical (3 categories) | Ordinal regression | 11.7 |
| Maternal pregnancy E-DII | Continuous | Predictive mean matching | 18.5 |
| Maternal pregnancy DASH | Continuous | Predictive mean matching | 5.7 |
| Maternal prepregnancy E-DII | Continuous | Predictive mean matching | 13.8 |
| Maternal prepregnancy DASH | Continuous | Predictive mean matching | 0.9 |
| Maternal sports physical activity | Binary | Logistic regression | 2.9 |
| Maternal leisure physical activity | Continuous | Predictive mean matching | 2 |
| Maternal work physical activity | Continuous | Predictive mean matching | 3.3 |
| Breastfeeding duration | Continuous | Predictive mean matching | 3.6 |
| Length of public bus lines within a 300-m buffer during pregnancy | Continuous | Predictive mean matching | 55.7 |
| Number of public bus stops within a 500-m buffer during pregnancy | Continuous | Predictive mean matching | 55.3 |
| Straight line distance to nearest blue space > 5,000 m^2^ during pregnancy | Continuous | Predictive mean matching | 27.3 |
| Green space > 5,000 m^2^ within a distance of 300 m during pregnancy | Binary | Logistic regression | 27.3 |
| Blue space > 5,000 m^2^ within a distance of 300 m at pregnancy | Binary | Logistic regression | 27.3 |
| Building density within a buffer of 300 m at pregnancy | Continuous | Predictive mean matching | 4.1 |
| Average of Normalized Difference Vegetation Index values within a buffer of 300 m at pregnancy | Continuous | Predictive mean matching | 3.9 |
| Percentage of land use for green urban areas and sports and leisure facilities within a 300-m buffer during pregnancy | Continuous | Predictive mean matching | 25.7 |
| Psychiatric disorder in pregnancy |  |  | 0 |
| Centre |  |  | 0 |

**Supplementary Table 4: List of variables included for imputation and % of missing data in Generation R**

| Variable | Type of variable | Model used to predict missing data | Missing value % |
| --- | --- | --- | --- |
| Maternal age | Continuous | Predictive mean matching | 0.03 |
| Paternal age | Continuous | Predictive mean matching | 17.7 |
| Parity mother | Binary | Logistic regression | 2.1 |
| Employment status mother | Binary | Logistic regression | 25.3 |
| Employment status father | Binary | Logistic regression | 45.6 |
| Education level mother | Categorical | Ordinal regression | 6.9 |
| Education level father | Categorical | Ordinal regression | 41.4 |
| Mother lives abroad | Binary | Logistic regression | 3.5 |
| Father lives abroad | Binary | Logistic regression | 10.8 |
| Parents live together | Binary | Logistic regression | 6.5 |
| Household income | Categorical | Ordinal regression | 26.0 |
| Food facility density | Binary | Logistic regression | 12.5 |
| Facility richness | Continuous | Predictive mean matching | 12.5 |
| Road and rail traffic | Continuous | Predictive mean matching | 3.2 |
| Street connectivity density | Continuous | Predictive mean matching | 12.8 |
| Population density | Continuous | Predictive mean matching | 12.8 |
| Area level socioeconomic indicator | Categorical | Ordinal regression | 12.8 |
| Access to green space | Continuous | Predictive mean matching | 12.8 |
| Psychiatric disorders during pregnancy | Binary | Logistic regression | 26.0 |
| Maternal prepregnancy BMI | Continuous | Predictive mean matching | 17.8 |
| Paternal pregnancy BMI | Continuous | Predictive mean matching | 29.5 |
| Maternal smoking during pregnancy | Categorical | Ordinal regression | 18.0 |
| Paternal smoking during pregnancy | Categorical | Ordinal regression | 16.4 |
| Gestational weight gain | Continuous | Predictive mean matching | 21.7 |
| Maternal DASH | Continuous | Predictive mean matching | 28.7 |
| Maternal DII | Continuous | Predictive mean matching | 28.7 |
| Breastfeeding | Continuous | Predictive mean matching | 44.5 |

**Supplementary Table 5: List of variables included for imputation and % of missing data in Lifeways**

| Variable | Type of variable | Model used to predict missing data | Missing value % |
| --- | --- | --- | --- |
| Maternal age | Continuous | Linear regression (pmm) | 0.6 |
| Paternal age | Continuous | Linear regression (pmm) | 13.7 |
| Maternal employment status | Binary | Logistic regression | 1.0 |
| Paternal employment status | Binary | Logistic regression | 18.7 |
| Parity | Binary | Logistic regression | 1.5 |
| Maternal education | Categorical (3 categories) | Ordinal regression | 2.4 |
| Paternal education | Categorical (3 categories) | Ordinal regression | 11.9 |
| Cohabitation status | Binary | Logistic regression | 0.1 |
| Household income | Binary | Logistic regression | 9.4 |
| Maternal prepregnancy BMI | Continuous | Linear regression (pmm) | 17.7 |
| Paternal BMI | Continuous | Linear regression (pmm) | 71.4 |
| Maternal smoking prepregnancy | Binary | Logistic regression | 17.2 |
| Paternal smoking prepregnancy | Binary | Logistic regression | 73.5 |
| Maternal smoking during pregnancy | Binary | Logistic regression | 4.6 |
| Paternal smoking during pregnancy | Binary | Logistic regression | 71.1 |
| Maternal pregnancy E-DII | Continuous | Linear regression (pmm) | 0.4 |
| Maternal pregnancy DASH | Continuous | Linear regression (pmm) | 0.4 |
| Paternal E-DII | Continuous | Linear regression (pmm) | 68.0 |
| Maternal physical activity level | Continuous | Linear regression (pmm) | 18.2 |
| Paternal physical activity level | Continuous | Linear regression (pmm) | 70.6 |
| Maternal psychiatric disorder during pregnancy (Moderately or extremely anxious or depressed) | Binary | Logistic regression | 2.5 |
| Maternal health coverage | Binary | Logistic regression | 0.2 |
| Number of antenatal visits (dichotomized) | Binary | Logistic regression | 45.5 |
| Child's sex | Binary | Logistic regression | 0.4 |
| (Any) Breastfeeding duration | Continuous | Linear regression (pmm) | 65.7 |

**Supplementary Table 6: Betas (95% CI) from imputed hierarchical multivariable linear regression analyses with maternal lifestyle patterns as the dependent variables. The EDEN study.** (N=1925)

| **Maternal lifestyle pattern 1: low smoking, high-quality diet and leisure PA** | | | | | | |
| --- | --- | --- | --- | --- | --- | --- |
|  | **Model 1** |  | **Model 2** |  | **Model 3** |  |
|  | β (95% CI) | P-value | β (95% CI) | P-value | β (95% CI) | P-value |
| **Socioeconomic and demographic characteristics** |  |  |  |  |  |  |
| **Centre** |  |  |  |  |  |  |
| Poitiers | ref | ref |  |  |  |  |
| Nancy | -0.04 (-0.16, 0.07) | 0.47 | -0.06 (-0.18, 0.07) | 0.38 | -0.09 (-0.22, 0.04) | 0.19 |
| **Maternal education level** |  |  |  |  |  |  |
| High | ref | ref |  |  |  |  |
| Medium | **-0.34 (-0.48, -0.20)** | **<.001** | -0.34 (-0.48, -0.19) | <.001 | -0.31 (-0.46, -0.17) | <.001 |
| Low | **-0.85 (-1.10, -0.60)** | **<.001** | -0.85 (-1.10, -0.60) | <.001 | -0.78 (-1.04, -0.53) | <.001 |
| **Maternal employment** |  |  |  |  |  |  |
| Employed/self-employed | ref | ref |  |  |  |  |
| Not employed | **-0.26 (-0.41, -0.11)** | **<.001** | -0.25 (-0.40, -0.09) | 0.002 | -0.23 (-0.38, -0.07) | 0.004 |
| **Parity** |  |  |  |  |  |  |
| Primiparous | ref | ref |  |  |  |  |
| Multiparous | **-0.18 (-0.31, -0.06)** | **0.004** | -0.18 (-0.31, -0.06) | 0.004 | -0.07 (-0.22, 0.07) | 0.30 |
| **Household income** |  |  |  |  |  |  |
| 4th quartile (highest) | ref | ref |  |  |  |  |
| 3rd quartile | -0.09 (-0.26, 0.07) | 0.26 | -0.10 (-0.26, 0.07) | 0.25 | -0.10 (-0.26, 0.06) | 0.23 |
| 2nd quartile | -0.12 (-0.30, 0.05) | 0.17 | -0.12 (-0.30, 0.06) | 0.18 | -0.12 (-0.29, 0.06) | 0.20 |
| 1st quartile | **-0.28 (-0.52, -0.03)** | **0.03** | -0.28 (-0.52, -0.03) | 0.03 | -0.22 (-0.47, 0.03) | 0.08 |
| **Parents live together** |  |  |  |  |  |  |
| Yes | ref | ref |  |  |  |  |
| No | 0.09 (-0.18, 0.36) | 0.51 | 0.10 (-0.17, 0.38) | 0.46 | 0.12 (-0.16, 0.39) | 0.40 |
| **Maternal age** | **0.06 (0.04, 0.07)** | **<.001** | 0.06 (0.04, 0.07) | <.001 | 0.05 (0.04, 0.07) | <.001 |
| **Mother born abroad** |  |  |  |  |  |  |
| No | ref | ref |  |  |  |  |
| Yes | **0.28 (0.00, 0.57)** | **0.05** | 0.28 (-0.02, 0.57) | 0.06 | 0.30 (0.00, 0.59) | 0.05 |
| **Urban environment** |  |  |  |  |  |  |
| **Access to green space** |  |  | -0.06 (-0.13, 0.01) | 0.11 | -0.06 (-0.13, 0.01) | 0.12 |
| **Population density** |  |  | 0.08 (0.00, 0.17) | 0.06 | 0.08 (-0.01, 0.17) | 0.08 |
| **Road and rail traffic** |  |  | 0.00 (-0.10, 0.10) | 0.93 | 0.01 (-0.09, 0.11) | 0.87 |
| **Street connectivity density** |  |  | 0.00 (-0.09, 0.09) | 0.97 | 0.00 (-0.09, 0.09) | 0.97 |
| **Food facility density** |  |  |  |  |  |  |
| Slightly unhealthy environment |  |  | ref | ref |  |  |
| Highly unhealthy environment |  |  | -0.08 (-0.25, 0.08) | 0.33 | -0.08 (-0.25, 0.08) | 0.33 |
| **Facility richness** |  |  |  |  |  |  |
| 0 |  |  | 0.02 (-0.20, 0.24) | 0.85 | 0.03 (-0.19, 0.25) | 0.81 |
| 0-0.05 |  |  | -0.01 (-0.19, 0.17) | 0.91 | -0.01 (-0.19, 0.17) | 0.92 |
| > 0.05 |  |  | ref | ref |  |  |
| **Area-level socioeconomic indicator during pregnancy** |  |  |  |  |  |  |
| Low level of deprivation |  |  |  | ref |  |  |
| Medium-low level of deprivation |  |  | -0.03 (-0.20, 0.14) | 0.69 | -0.03 (-0.20, 0.14) | 0.70 |
| Medium level of deprivation |  |  | -0.12 (-0.31, 0.07) | 0.20 | -0.13 (-0.32, 0.06) | 0.18 |
| Medium-high level of deprivation |  |  | -0.07 (-0.26, 0.12) | 0.47 | -0.06 (-0.25, 0.13) | 0.56 |
| High level of deprivation |  |  | -0.12 (-0.32, 0.08) | 0.24 | -0.10 (-0.30, 0.10) | 0.31 |
| **Psychosocial factors and health-care access** |  |  |  |  |  |  |
| **Psychiatric disorders during pregnancy** |  |  |  |  |  |  |
| No |  |  |  |  | ref | ref |
| Yes |  |  |  |  | 0.04 (-0.21, 0.28) | 0.78 |
| **Free/subsidised health insurance for very low-income families or no complementary insurance** |  |  |  |  |  |  |
| No |  |  |  |  | ref | ref |
| Yes |  |  |  |  | -0.11 (-0.34, 0.11) | 0.32 |
| **Antenatal visits** |  |  |  |  |  |  |
| < 7 |  |  |  |  | -0.07 (-0.25, 0.11) | 0.44 |
| ≥ 7 |  |  |  |  | ref | ref |
| **Antenatal preparation for parenting** |  |  |  |  |  |  |
| No |  |  |  |  | **-0.23 (-0.38, -0.08)** | **0.003** |
| Yes for all |  |  |  |  | ref | ref |
| Yes for some |  |  |  |  | -0.11 (-0.28, 0.05) | 0.18 |
|  |  |  |  |  |  |  |
| **Maternal lifestyle pattern 2: low BMI and high GWG** | | | | | | |
|  | | | | | | |
|  | **Model 1** |  | **Model 2** |  | **Model 3** |  |
|  | β (95% CI) | P-value | β (95% CI) | P-value | β (95% CI) | P-value |
| **Socioeconomic and demographic characteristics** |  |  |  |  |  |  |
| **Centre** |  |  |  |  |  |  |
| Poitiers | ref | ref | ref | ref |  |  |
| Nancy | -0.01 (-0.11, 0.09) | 0.82 | -0.04 (-0.15, 0.07) | 0.49 | -0.01 (-0.12, 0.10) | 0.84 |
| **Maternal education level** |  |  |  |  |  |  |
| High | ref | ref | ref | ref |  |  |
| Medium | 0.07 (-0.06, 0.20) | 0.28 | 0.08 (-0.05, 0.21) | 0.21 | 0.08 (-0.04, 0.21) | 0.19 |
| Low | **0.24 (0.02, 0.46)** | **0.03** | 0.25 (0.03, 0.47) | 0.03 | 0.30 (0.08, 0.52) | 0.008 |
| **Maternal employment** |  |  |  |  |  |  |
| Employed/self-employed | ref | ref |  |  |  |  |
| Not employed | **-0.30 (-0.43, -0.17)** | **<.001** | -0.30 (-0.43, -0.17) | <.001 | -0.28 (-0.42, -0.15) | <.001 |
| **Parity** |  |  |  |  |  |  |
| Primiparous | ref | ref | ref | ref |  |  |
| Multiparous | **-0.27 (-0.38, -0.16)** | **<.001** | -0.26 (-0.37, -0.15) | <.001 | -0.22 (-0.35, -0.10) | <.001 |
| **Household income** |  |  |  |  |  |  |
| 4th quartile (highest) | ref | ref |  |  |  |  |
| 3rd quartile | -0.12 (-0.26, 0.02) | 0.10 | -0.11 (-0.25, 0.03) | 0.12 | -0.11 (-0.26, 0.03) | 0.11 |
| 2nd quartile | **-0.31 (-0.46, -0.16)** | **<.001** | -0.31 (-0.46, -0.16) | <.001 | -0.32 (-0.47, -0.16) | <.001 |
| 1st quartile (lowest) | **-0.29 (-0.50, -0.08)** | **0.007** | -0.28 (-0.49, -0.07) | 0.01 | -0.24 (-0.45, -0.02) | 0.03 |
| **Parents live together** |  |  |  |  |  |  |
| Yes | ref | ref |  |  |  |  |
| No | -0.06 (-0.29, 0.18) | 0.62 | -0.05 (-0.28, 0.19) | 0.70 | -0.03 (-0.04, -0.02) | <.001 |
| **Maternal age** | **-0.03 (-0.04, -0.02)** | **<.001** | -0.03 (-0.04, -0.02) | <.001 | -0.01 (-0.03, 0.01) | 0.20 |
| **Mother born abroad** |  |  |  |  |  |  |
| No | ref | ref |  |  |  |  |
| Yes | -0.19 (-0.44, 0.06) | 0.13 | -0.21 (-0.46, 0.05) | 0.11 | -0.21 (-0.47, 0.04) | 0.10 |
| **Urban environment** |  |  |  |  |  |  |
| **Access to green space** |  |  | -0.02 (-0.08, 0.04) | 0.54 | -0.02 (-0.08, 0.04) | 0.58 |
| **Population density** |  |  | **0.09 (0.02, 0.16)** | **0.02** | 0.09 (0.02, 0.17) | 0.02 |
| **Road and rail traffic** |  |  | -0.01 (-0.09, 0.07) | 0.74 | -0.01 (-0.10, 0.07) | 0.73 |
| **Street connectivity density** |  |  | -0.03 (-0.11, 0.05) | 0.46 | -0.03 (-0.11, 0.05) | 0.52 |
| **Food facility density** |  |  |  |  |  |  |
| Slightly unhealthy environment |  |  | ref | ref |  |  |
| Highly unhealthy environment |  |  | 0.05 (-0.10, 0.19) | 0.53 | 0.05 (-0.09, 0.19) | 0.50 |
| **Facility richness** |  |  |  |  |  |  |
| 0 |  |  | -0.09 (-0.28, 0.10) | 0.36 | -0.08 (-0.27, 0.11) | 0.42 |
| 0-0.05 |  |  | -0.11 (-0.27, 0.05) | 0.18 | -0.10 (-0.26, 0.06) | 0.21 |
| > 0.05 |  |  | ref | ref |  |  |
| **Area-level socioeconomic indicator during pregnancy** |  |  |  |  |  |  |
| Low level of deprivation |  |  | ref | ref |  |  |
| Medium-low level of deprivation |  |  | -0.05 (-0.20, 0.10) | 0.49 | -0.05 (-0.20, 0.09) | 0.47 |
| Medium level of deprivation |  |  | 0.00 (-0.16, 0.16) | 0.99 | 0.00 (-0.16, 0.16) | 0.99 |
| Medium-high level of deprivation |  |  | -0.12 (-0.28, 0.04) | 0.15 | -0.11 (-0.27, 0.05) | 0.19 |
| High level of deprivation |  |  | **-0.21 (-0.38, -0.04)** | **0.02** | -0.20 (-0.37, -0.02) | 0.03 |
| **Psychosocial factors and health-care access** |  |  |  |  |  |  |
| **Psychiatric disorders during pregnancy** |  |  |  |  |  |  |
| No |  |  |  |  | ref | ref |
| Yes |  |  |  |  | **-0.38 (-0.59, -0.17)** | **<.001** |
| **Free/subsidised health insurance for very low-income families** |  |  |  |  |  |  |
| No |  |  |  |  | ref | ref |
| Yes |  |  |  |  | **-0.20 (-0.40, 0.00)** | **0.05** |
| **Antenatal visits** |  |  |  |  |  |  |
| < 7 |  |  |  |  | 0.14 (-0.02, 0.29) | 0.09 |
| ≥ 7 |  |  |  |  | ref | ref |
| **Antenatal preparation for parenting** |  |  |  |  |  |  |
| No |  |  |  |  | -0.07 (-0.20, 0.06) | 0.28 |
| Yes for all |  |  |  |  | ref | ref |
| Yes for some |  |  |  |  | -0.07 (-0.21, 0.07) | 0.31 |

PA: physical activity. Model 1 included maternal socioeconomic and demographic factors and additional adjustment for paternal education level, model 2 urban environmental factors, and model 3 psychosocial factors and access to health care. For the sake of parsimony, the effect of each variable was adjusted for the other variables from the same block, and additionally adjusted for variables from the preceding block. Coefficients are interpreted when the variable appear the first time but we showed effects for further models for indication.

**Supplementary Table 7: Betas (95% CI) from imputed hierarchical multivariable linear regression analyses with maternal lifestyle patterns as the dependent variables. Generation R study.** (N=8546)

| **Maternal lifestyle pattern 1: high BMI, smoking, poor-quality diet** | | | | | | | |
| --- | --- | --- | --- | --- | --- | --- | --- |
|  |  | **Model 1** |  | **Model 2** |  | **Model 3** |  |
|  |  | β (95% CI) | P-value | β (95% CI) | P-value | β (95% CI) | P-value |
| **Socioeconomic and demographic characteristics** |  |  |  |  |  |  |  |
| **Maternal education level** |  |  |  |  |  |  |  |
| High |  | ref | ref | ref | ref | ref | ref |
| Medium |  | **0.42 (0.35, 0.49)** | **<.001** | 0.41 (0.34, 0.48) | <.001 | 0.41 (0.34, 0.48) | <.001 |
| Low |  | **0.47 (0.36, 0.58)** | **<.001** | 0.46 (0.35, 0.57) | <.001 | 0.46 (0.35, 0.57) | <.001 |
| **Paternal education** |  |  |  |  |  |  |  |
| High |  | ref | ref | ref | ref | ref | ref |
| Medium |  | **0.25 (0.18, 0.32)** | **<.001** | 0.25 (0.17, 0.32) | <.001 | 0.25 (0.17, 0.32) | <.001 |
| Low |  | **0.42 (0.28, 0.55)** | **<.001** | 0.41 (0.28, 0.54) | <.001 | 0.41 (0.27, 0.54) | <.001 |
| **Maternal employment** |  |  |  |  |  |  |  |
| Employed/self-employed |  | ref | ref | ref | ref | ref | ref |
| Not employed |  | **0.12 (0.04, 0.19)** | **0,004** | 0.12 (0.04, 0.19) | 0.004 | 0.11 (0.04, 0.19) | 0.005 |
| **Parity** |  |  |  |  |  |  |  |
| Primiparous |  | ref | ref | ref | ref | ref | ref |
| Multiparous |  | **0.34 (0.28, 0.40)** | **<.001** | 0.33 (0.27, 0.38) | <.001 | 0.33 (0.27, 0.38) | <.001 |
| **Household income** |  |  |  |  |  |  |  |
| 4th quartile (highest) > 2,200 |  | ref | ref | ref | ref | ref | ref |
| 3rd quartile (1,200-2,200) |  | **0.12 (0.04, 0.19)** | **0.002** | 0.12 (0.04, 0.19) | 0.002 | 0.12 (0.04, 0.19) | 0.002 |
| 2nd quartile <= 1,200 |  | **0.16 (0.06, 0.26)** | **0.002** | 0.15 (0.05, 0.25) | 0.003 | 0.15 (0.05, 0.25) | 0.003 |
| 1st quartile |  | NA |  |  |  |  |  |
| **Parents live together** |  |  |  |  |  |  |  |
| Yes |  | ref | ref | ref | ref | ref | ref |
| No |  | **0.16 (0.07, 0.26)** | **<.001** | 0.16 (0.07, 0.26) | <.001 | 0.16 (0.07, 0.25) | <.001 |
| **Maternal age** |  | **-0.03 (-0.03, -0.02)** | **<.001** | -0.03 (-0.03, -0.02) | <.001 | -0.03 (-0.03, -0.02) | <.001 |
| **Mother born abroad** |  |  |  |  |  |  |  |
| No |  | ref | ref | ref | ref | ref | ref |
| Yes |  | **-0.17 (-0.23, -0.11)** | **<.001** | -0.17 (-0.24, -0.11) | <.001 | -0.17 (-0.24, -0.11) | <.001 |
| **Urban environment** |  |  |  |  |  |  |  |
| **Population density** |  |  |  | 0.03 (0.00, 0.06) | 0.06 | 0.03 ( 0.00, 0.06) | 0.06 |
| **Access to green space** |  |  |  | 0.01 (-0.02, 0.04) | 0.45 | 0.01 (-0.02, 0.04) | 0.45 |
| **Road and rail traffic** |  |  |  | -0.03 (-0.06, 0.01) | 0.10 | -0.03 (-0.06, 0.01) | 0.10 |
| **Street connectivity density** |  |  |  | -0.01 (-0.05, 0.02) | 0.49 | -0.01 (-0.05, 0.02) | 0.49 |
| **Food facility density** |  |  |  |  |  |  |  |
| Slightly unhealthy environment |  |  |  | ref | ref | ref | ref |
| Highly unhealthy environment |  |  |  | -0.03 (-0.12, 0.07) | 0.57 | -0.03 (-0.12, 0.07) | 0.56 |
| **Facility richness** |  |  |  | -0.02 (-0.06, 0.01) | 0.24 | -0.02 (-0.06, 0.01) | 0.24 |
| **Area-level socioeconomic during pregnancy** |  |  |  |  |  |  |  |
| Low level of deprivation |  |  |  | **ref** | **ref** | **ref** | **ref** |
| Medium-low level of deprivation |  |  |  | -0.09 (-0.24, 0.05) | 0.20 | -0.09 (-0.24, 0.05) | 0.20 |
| Medium level of deprivation |  |  |  | -0.02 (-0.15, 0.10) | 0.72 | -0.02 (-0.15, 0.10) | 0.72 |
| Medium-high level of deprivation |  |  |  | -0.02 (-0.17, 0.12) | 0.77 | -0.02 (-0.17, 0.12) | 0.77 |
| High level of deprivation |  |  |  | 0.03 (-0.09, 0.15) | 0.64 | 0.03 (-0.10, 0.15) | 0.64 |
| **Psychosocial factors and access to health care** | |  |  |  |  |  |  |
| **Psychiatric disorders during pregnancy** | |  |  |  |  |  |  |
| No |  |  |  |  |  | **ref** | **ref** |
| Yes |  |  |  |  |  | 0.03 (-0.06, 0.13) | 0.52 |
|  |  |  |  |  |  |  |  |
| **Maternal lifestyle pattern 2: low BMI, high GWG, and smoking** | | | | | | | |
|  |  |  |  |  |  |  |  |
|  |  | **Model 1** |  | **Model 2** |  | **Model 3** |  |
|  |  | β (95% CI) | P-value | β (95% CI) | P-value | β (95% CI) | P-value |
| **Socioeconomic and demographic characteristics** |  |  |  |  |  |  |  |
| **Maternal education level** |  |  |  |  |  |  |  |
| High |  | **ref** | **ref** | **ref** | **ref** | **ref** | **ref** |
| Medium |  | 0.02 (-0.04, 0.08) | 0.52 | 0.03 (-0.04, 0.09) | 0.40 | 0.03 (-0.04, 0.09) | 0.42 |
| Low |  | -0.01 (-0.11, 0.09) | 0.83 | -0.01 (-0.10, 0.09) | 0.91 | -0.01 (-0.11, 0.09) | 0.90 |
| **Paternal education** |  |  |  |  |  |  |  |
| High |  | ref | ref | ref | ref | ref | ref |
| Medium |  | 0.04 (-0.02, 0.11) | 0.17 | 0.05 (-0.02, 0.11) | 0.14 | 0.05 (-0.02, 0.11) | 0.14 |
| Low |  | 0.03 (-0.07, 0.14) | 0.52 | 0.04 (-0.07, 0.14) | 0.47 | 0.04 (-0.07, 0.14) | 0.50 |
| **Maternal employment** |  |  |  |  |  |  |  |
| Employed/self-employed |  | ref | ref | ref | ref | ref | ref |
| Not employed |  | 0.07 (0.00, 0.14) | 0.06 | 0.06 (-0.01, 0.13) | 0.08 | 0.06 (-0.01, 0.13) | 0.10 |
| **Parity** |  |  |  |  |  |  |  |
| Primiparous |  | ref | ref | ref | ref | ref | ref |
| Multiparous |  | **-0.20 (-0.25, -0.15)** | **<.001** | -0.19 (-0.24, -0.14) | <.001 | -0.19 (-0.24, -0.14) | <.001 |
| **Household income** |  |  |  |  |  |  |  |
| 4th quartile (highest) > 2,200 |  | ref | ref | ref | ref | ref | ref |
| 3rd quartile (1,200-2,200) |  | 0.03 (-0.04, 0.10) | 0.46 | 0.03 (-0.04, 0.10) | 0.41 | 0.03 (-0.04, 0.10) | 0.44 |
| 2nd quartile <= 1,200 |  | -0.01 (-0.10, 0.09) | 0.87 | 0.00 (-0.10, 0.09) | 0.95 | -0.01 (-0.10, 0.09) | 0.85 |
| 1st quartile |  | NA |  |  |  |  |  |
| **Parents live together** |  |  |  |  |  |  |  |
| Yes |  | ref | ref | ref | ref | ref | ref |
| No |  | **0.25 (0.17, 0.33)** | **<.001** | 0.25 (0.18, 0.33) | <.001 | 0.25 (0.17, 0.33) | <.001 |
| **Maternal age** |  | 0.00 (-0.01, 0.00) | 0.46 | 0.00 (-0.01, 0.00) | 0.41 | 0.00 (-0.01, 0.00) | 0.47 |
| **Mother born abroad** |  |  |  |  |  |  |  |
| No |  | ref | ref | ref | ref | ref | ref |
| Yes |  | **-0.32 (-0.38, -0.27)** | **<.001** | -0.32 (-0.38, -0.27) | <.001 | -0.33 (-0.38, -0.27) | <.001 |
| **Urban environment** |  |  |  |  |  |  |  |
| **Population density** |  |  |  | -0.01 (-0.04, 0.01) | 0.26 | -0.01 (-0.04, 0.01) | 0.27 |
| **Access to green space** |  |  |  | -0.01 (-0.03, 0.02) | 0.59 | -0.01 (-0.03, 0.02) | 0.59 |
| **Road and rail traffic** |  |  |  | 0.00 (-0.03, 0.03) | 0.97 | 0.00 (-0.03, 0.03) | 0.96 |
| **Street connectivity density** |  |  |  | **0.04 (0.01, 0.07)** | **0.01** | 0.04 (0.01, 0.07) | 0.01 |
| **Food facility density** |  |  |  |  |  |  |  |
| Slightly unhealthy environment |  |  |  | ref | ref | ref | ref |
| Highly unhealthy environment |  |  |  | 0.01 (-0.07, 0.09) | 0.82 | 0.01 (-0.07, 0.09) | 0.83 |
| **Facility richness** |  |  |  | 0.02 (-0.02, 0.05) | 0.38 | 0.02 (-0.02, 0.05) | 0.38 |
| **Area-level socioeconomic indicator during pregnancy** | | |  |  |  |  |  |
| Low level of deprivation |  |  |  | ref | ref | ref | ref |
| Medium-low level of deprivation |  |  |  | -0.05 (-0.17, 0.08) | 0.45 | -0.05 (-0.17, 0.08) | 0.45 |
| Medium level of deprivation |  |  |  | -0.05 (-0.17, 0.06) | 0.35 | -0.05 (-0.17, 0.06) | 0.35 |
| Medium-high level of deprivation |  |  |  | -0.05 (-0.18, 0.08) | 0.46 | -0.05 (-0.18, 0.08) | 0.46 |
| High level of deprivation |  |  |  | -0.09 (-0.20, 0.02) | 0.11 | -0.09 (-0.20, 0.02) | 0.11 |
| **Psychiatric disorders during pregnancy** | |  |  |  |  |  |  |
| No |  |  |  |  |  | ref | ref |
| Yes |  |  |  |  |  | 0.07 (-0.03, 0.16) | 0.18 |

Model 1 included maternal socioeconomic and demographic factors and additional adjustment for paternal education level, model 2 urban environmental factors, and model 3 psychosocial factors and access to health care. For the sake of parsimony, the effect of each variable was adjusted for the other variables from the same block, and additionally adjusted for variables from the preceding block. Coefficients are interpreted when the variable appear the first time but we showed effects for further models for indication.

**Supplementary Table 8: Betas (95% CI) from imputed hierarchical multivariable linear regression analyses with maternal lifestyle patterns as the dependent variables. The Lifeways study.** (N=931)

| **Maternal lifestyle 1: Smoking and poor-quality diet** | | | | | |
| --- | --- | --- | --- | --- | --- |
|  |  | **Model 1** |  | **Model 3** |  |
|  |  | β (95% CI) | P-value | β (95% CI) | P-value |
| **Socioeconomic and demographic factors** |  |  |  |  |  |
| **Maternal education level** |  |  |  |  |  |
| High |  | ref | ref | ref | ref |
| Medium |  | **0.40 (0.21, 0.59)** | **<.001** | 0.33 (0.14, 0.52) | <.001 |
| Low |  | **0.67 (0.43, 0.90)** | **<.001** | 0.58 (0.34, 0.81) | <.001 |
| **Paternal education** |  |  |  |  |  |
| High |  | ref | ref | ref | ref |
| Medium |  | **0.25 (0.05, 0.46)** | **0.02** | 0.21 (-0.01, 0.42) | 0.06 |
| Low |  | **0.29 (0.08, 0.49)** | **0.001** | 0.19 (-0.02, 0.39) | 0.07 |
| **Maternal employment** |  |  |  |  |  |
| Employed/self-employed |  | ref | ref | ref | ref |
| No employed |  | -0.08 (-0.27, 0.10) | 0.37 | -0.14 (-0.33, 0.04) | 0.13 |
| **Parity** |  |  |  |  |  |
| Primiparous |  | ref | ref | ref | ref |
| Multiparous |  | **0.28 (0.10, 0.47)** | **0.003** | 0.29 (0.11, 0.47) | 0.002 |
| **Household income** |  |  |  |  |  |
| 4th quartile (highest) ≥600£/week |  | ref | ref | ref | ref |
| 3rd quartile |  | n.a. | n.a. | n.a. | n.a. |
| 2nd quartile |  | n.a. | n.a. | n.a. | n.a. |
| 1st quartile (lowest) <600£/week |  | **0.30 (0.12, 0.48)** | **0.001** | 0.23 (0.05, 0.41) | 0.01 |
| **Parents live together** |  |  |  |  |  |
| Yes |  | ref | ref | ref | ref |
| No |  | **0.22 (0.02, 0.42)** | **0.03** | 0.18 (-0.01, 0.38) | 0.07 |
| **Maternal age** |  | **-0.07 (-0.08, -0.05)** | **<.001** | -0.06 (-0.07, -0.04) | <.001 |
| **Psychosocial factors and access to health care** | |  |  |  |  |
| **Psychiatric disorders during pregnancy** | |  |  |  |  |
| No |  |  |  | ref | ref |
| Yes |  |  |  | 0.17 (-0.02, 0.36) | 0.08 |
| **Free/subsidised health insurance for very low-income families** | |  |  |  |  |
| No |  |  |  | ref | ref |
| Yes |  |  |  | **0.39 (0.21, 0.57)** | **<.001** |
| **Antenatal visits** |  |  |  |  |  |
| <6 antenatal visits |  |  |  | 0.14 (-0.09, 0.37) | 0.24 |
| ≥6 antenatal visits |  |  |  | ref | ref |
| **Maternal lifestyle 2: Low BMI and high PA** | | | | | |
|  |  | **Model 1** |  | **Model 3** |  |
|  |  | β (95% CI) | P-value | β (95% CI) | P-value |
| **Socioeconomic and demographic factors** |  |  |  |  |  |
| **Maternal education level** |  |  |  |  |  |
| High |  | ref | ref | ref | ref |
| Medium |  | 0.03 (-0.13, 0.19) | 0.72 | 0.04 (-0.13, 0.20) | 0.68 |
| Low |  | 0.04 (-0.16, 0.25) | 0.67 | 0.05 (-0.16, 0.25) | 0.67 |
| **Paternal education** |  |  |  |  |  |
| High |  | ref | ref | ref | ref |
| Medium |  | 0.01 (-0.17, 0.19) | 0.91 | -0.02 (-0.20, 0.17) | 0.86 |
| Low |  | -0.11 (-0.29, 0.07) | 0.23 | -0.11 (-0.30, 0.07) | 0.24 |
| **Maternal employment** |  |  |  |  |  |
| Employed/self-employed |  | ref | ref | ref | ref |
| Not employed |  | 0.10 (-0.06, 0.26) | 0.23 | 0.10 (-0.07, 0.26) | 0.25 |
| **Parity** |  |  |  |  |  |
| Primiparous |  | ref | ref | ref | ref |
| Multiparous |  | -0.14 (-0.29, 0.02) | 0.09 | -0.15 (-0.31, 0.01) | 0.07 |
| **Household income** |  |  |  |  |  |
| 4th quartile (highest) ≥600£/week |  | ref | ref | ref | ref |
| 3rd quartile |  | n.a. | n.a. | n.a. | n.a. |
| 2nd quartile |  | n.a. | n.a. | n.a. | n.a. |
| 1st quartile (lowest) <600£/week |  | **-0.17 (-0.32, -0.01)** | **0.03** | -0.16 (-0.32, -0.01) | 0.04 |
| **Parents live together** |  |  |  |  |  |
| Yes |  | ref | ref | ref | ref |
| No |  | 0.09 (-0.08, 0.25) | 0.32 | 0.09 (-0.08, 0.26) | 0.29 |
| **Maternal age** |  | **-0.02 (-0.03, -0.01)** | **0.01** | -0.02 (-0.03, 0.00) | 0.01 |
| **Psychosocial factors and access to health care** | |  |  |  |  |
| **Psychiatric disorders during pregnancy** | |  |  |  |  |
| No |  |  |  | ref | ref |
| Yes |  |  |  | -0.04 (-0.21, 0.13) | 0.65 |
| **Free/subsidised health insurance** | |  |  |  |  |
| No |  |  |  | ref | ref |
| Yes |  |  |  | -0.01 (-0.17, 0.15) | 0.90 |
| **Antenatal visits** |  |  |  |  |  |
| <6 antenatal visits |  |  |  | 0.19 (0.00, 0.39) | 0.06 |
| ≥6 antenatal visits |  |  |  | ref | ref |

Model 1 included maternal socio-economic and demographic factors and additional adjustment for paternal education level, variables for model 2 are not available for Lifeways, and model 3 psychosocial factors and access to health care. For the sake of parsimony, the effect of each variable was adjusted for the other variables from the same block, and additionally adjusted for variables from the preceding block. Coefficients are interpreted when the variable appear the first time but we showed effects for further models for indication.
